# The true performance of Li-CO_2_ batteries for resolving the disagreement on their feasibility

**DOI:** 10.1016/j.isci.2025.114047

**Published:** 2025-11-14

**Authors:** Kai Chen, Jia-Yi Du, Hao-Ran Zhang, Ying-Qi Fan, Dong-Yue Yang, Jin Wang, Kai Li, Gang Huang

**Affiliations:** 1 State Key Laboratory of Rare Earth Resource Utilization, Changchun Institute of Applied Chemistry, Chinese Academy of Sciences, Changchun, Jilin, China; 2 University of Science and Technology of China, Hefei, Anhui, China

**Keywords:** Applied sciences, Energy engineering, Energy systems

## Abstract

Lithium (Li)-CO_2_ batteries, capable of converting CO_2_ to Li_2_CO_3_/C while delivering significant energy, have attracted extensive research interest. However, their feasibility remains controversial and underexplored. To address this, a rigorous testing system combining ECC-air battery models and differential electrochemical mass spectrometry (DEMS) was developed to ensure a pure gas environment. Findings reveal that even minute O_2_ permeation (0.5%) from air can elevate the discharge plateau, misleadingly transforming Li-CO_2_ into Li-O_2_/CO_2_ batteries. Under strict testing, true Li-CO_2_ battery performance falls short of reported successes. Experimental and theoretical confirmation shows the 4Li + 3CO_2_ → 2Li_2_CO_3_ + C reaction proceeds at a lower discharge plateau (<2.0 V), a voltage window traditionally overlooked. This work underscores the need for rigorous testing to enhance scientific reliability in Li-CO_2_ battery research so that this field can develop sustainably.

## Highlights

• Developing a testing system to reveal the true performance of Li-CO_2_ batteries
• 0.5% O_2_ in CO_2_ could lift the discharge voltage of Li-CO_2_ batteries
• The reaction 4Li + 3CO_2_ → 2Li_2_CO_3_ + C is confirmed by many techniques
• Five strategies are proposed to help check the sealibility of testing systems

## Graphical abstract

**Figure:**
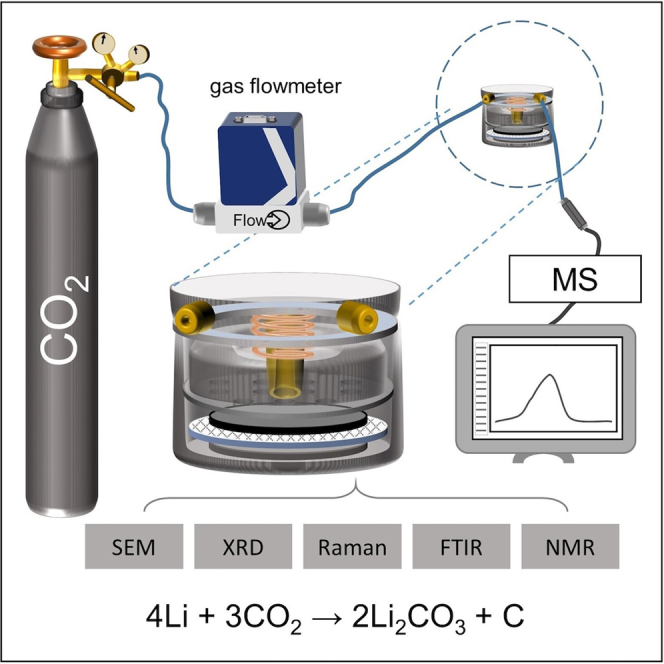

## Introduction

Carbon dioxide, as a major component from industrial gas emission, has raised greenhouse effect for nearly a century. In recent years, transforming CO_2_ into high-value chemical products is highly desirable in a variety of fields, such as energy, electrochemistry, (electro)-chemical synthesis, etc.^1^^,^^2^^,^^3^ Among the applications, Li-CO_2_ battery has received much attention because it combines the battery and greenhouse gas CO_2_, and it is also considered as an optimal battery system in Mars exploration, which consists 95% CO_2_, 2.8% N_2_, 1.6% Ar, 0.174% O_2_, and 0.074% CO.

During the past decade, many works have been conducted to improve the performance of Li-CO_2_ batteries and Li-CO_2 Mars_ batteries, but most of them have been focused on the cathode material design.^4^^,^^5^^,^^6^^,^^7^^,^^8^^,^^9^^,^^10^^,^^11^^,^^12^^,^^13^^,^^14^ Few works have touched the fundamental chemistries of Li-CO_2_ batteries, like the reaction mechanism during discharge, the key intermediates, and most importantly, the feasibility of Li-CO_2_ batteries. The first report on Li-CO_2_ battery came from Kensuke Takechi’s group in 2011.^15^ However, this report claimed that negligible capacity (66 mAh g^−1^) could be obtained from Li-CO_2_ battery with a KB cathode. In 2013, B. D. McCloskey reported similar results with XC72 cathode,^16^ and L.A.Archer realized Li-CO_2_ battery only at elevated temperatures (60°C, 80°C, and 100°C) while the capacity at 25°C was nearly zero.^17^ Moreover, recently, Lu revealed that CO_2_ was not electrochemically active in Li-CO_2_ batteries but the activity could be improved by H_2_O.^18^ Besides, Tan claimed that the operating voltage is ∼1.1V.^19^ The so-called successful Li-CO_2_ battery at room temperature was first raised by Y. Liu with KB cathode in 2014.^20^ In the next year, Zhou’s group adopted graphene and carbon nanotubes as cathodes and realized high-capacity Li-CO_2_ batteries.^4^^,^^5^ After that, the publications on Li-CO_2_ batteries boomed. During the surge of the research on Li-CO_2_ batteries, many groups still cannot repeat the results of Li-CO_2_ batteries with discharge plateaus above 2.5 V or large capacities even using different electrolytes and excellent catalysts.^3^^,^^16^^,^^18^^,^^21^^,^^22^^,^^23^^,^^24^ Until now, the reasons for the controversial results have not been resolved and no consensus have been reached on this topic. This debate has hindered basic understandings and sustainable development of Li-CO_2_ batteries, thus it is critical to conduct a thorough research on the feasibility of Li-CO_2_ batteries and uncover the hidden reasons for the disagreement.

We noticed that the experimental testing conditions in previous studies were simply described without key information such as testing bottle tightness and sealing check during the test.^4^^,^^25^^,^^26^^,^^27^^,^^28^^,^^29^^,^^30^^,^^31^^,^^32^^,^^33^^,^^34^ Poor tightness of the testing system could induce air (O_2_) permeation, which may influence the battery performance, including O_2_-involved electrochemical reactions that render a higher discharge voltage and larger capacity. Therefore, adopting rigorous testing systems is critical to reveal the true performance of Li-CO_2_ batteries and reconcile the long-lasting disagreement on their feasibility.

Here, we have adopted ECC-air battery models and differential electrochemical mass spectrometry (DEMS) to configure a rigorous testing system to ensure a pure gas environment. Under our strict experiment design and conduct, we have found the reported high performance of Li-CO_2_ batteries with high discharge plateaus (>2.5 V) and large discharge capacities cannot be reproduced. However, when a trace amount of O_2_ (as low as 0.5%) was introduced into the testing system, the performance (capacity and discharge voltage) could be significantly improved, thus we hypothesize that some reported Li-CO_2_ batteries are actually O_2_-involved Li-CO_2_ batteries because of the imperfect testing condition leading to O_2_ permaeation. Typically, 2.0 V is considered as the cutoff voltage in previous works while the true Li-CO_2_ batteries have minimal capacity above 2.0 V. However, when 1.0 V is set as the cutoff voltage, discharge plateau can be observed (∼1.5 V), and the capacity improves. After discharge, Li_2_CO_3_ and C can be detected on the carbon-free nanoporous gold cathode using Raman spectroscopy, confirming the viability of Li-CO_2_ batteries at lower discharge voltages. The CO_2_ consumption has also been monitored during discharge and it reveals a 1.39 e^−^/CO_2_ coefficient, consistent with the electron transfer per CO_2_ in the reaction of 4Li + 3CO_2_ → 2Li_2_CO_3_ + C. Additionally, Isotope labeling with ^13^CO_2_ has been adopted to verify the formation of Li_2_^13^CO_3_ and ^13^C by nuclear magnetic resonance (NMR). On these bases, we have experimentally confirmed that the equilibrium potential of Li-CO_2_ battery is 2.80 V, and the actual low discharge voltage during battery operation comes from the sluggish kinetics of CO_2_ reduction, which is explained by theoretical calculations. To guide future research on Li-CO_2_ batteries, five strategies have been proposed to help check the sealability of the testing systems to prevent unconscious O_2_ influence. Through our investigation, it can be concluded that Li-CO_2_ batteries are feasible but the sluggish kinetics lower the discharge voltage, which clarifies the long-lasting controversial problem.

## Results and discussion

### The influence of O_2_ on Li-CO_2_ batteries

As to the testing condition of Li-CO_2_ batteries, many reports sealed the assembled battery in a glass bottle, and then followed by gas flowing to fill the bottle with CO_2_. There were also some reports using a glove box filled with CO_2_ or pressurized gas. However, few discussions on how to ensure the sealability of the bottle or cell were revealed. Also, no details explained whether static gas or flowing gas was used during the test, and whether the contaminants from open air could permeate into the bottle was unknown. It should be kept in mind that even trace O_2_ permeation can cover the real performance of Li-CO_2_ batteries.^22^ Therefore, a rigorous experiment that could ensure gas purity is needed to reveal the true performance of Li-CO_2_ batteries.

**Figure 1:**
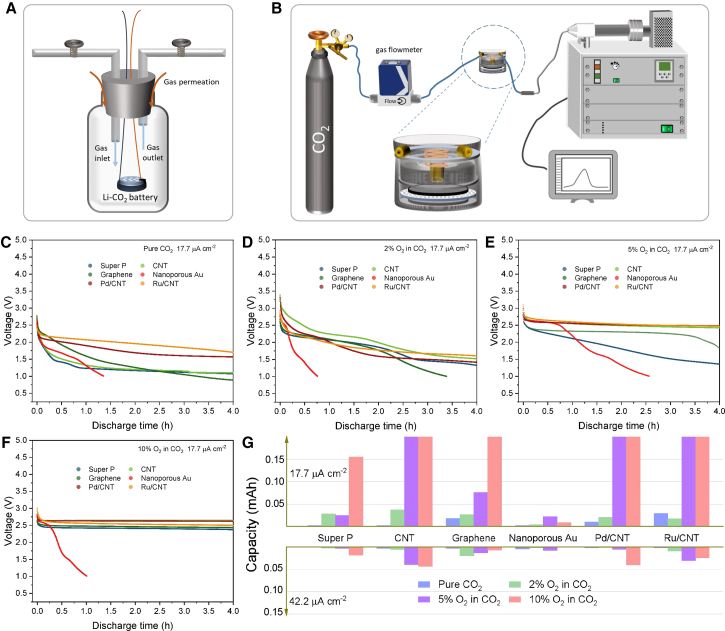
Test equipments and discharge performance (A) Typical testing configuration of Li-CO_2_ batteries in previous works. (B) The battery testing system used in this work. (C–F) Discharge performance of Li-CO_2_ batteries with different cathodes and 0% (C), 2% (D), 5% (E), 10% (F) O_2_ in CO_2_ at 17.7 μA cm^−2^. (G) Discharge capacities of Li-CO_2_ batteries with a cut-off voltage of 2.0 V.

Figure 1A gives the typical testing system of Li-CO_2_ batteries with an inlet and an outlet to expel the residue gas in the bottle. The drawbacks of this system are obvious. After gas purging, the bottle will be transferred to test in a thermotank, and the sealability of the bottle during the test cannot be guaranteed. The gas purity in the bottle is related to the gas-flow rate and time and the volume of the glass bottle. It is difficult to ensure the gas purity without quantifying the mentioned parameters or apparatus for composition analysis. Even though the gas purity is high initially, when the stress on the stopper is relieved, gas diffusion cannot be prevented even if the rate is slow. As a result, the air components, especially O_2_, can participate in the discharge reaction. To address the intrinsic flaws of the above testing system, we adopted a system with rigorous sealing and impurity test. In our experiment design, an ECC-air battery model with a gas volume of 4.2 mL in flowing gas was used to make a perfect CO_2_ environment during the test. The battery is connected to a gas flowmeter to control the flow rate at the inlet, and the outlet is connected to a mass spectrometer to monitor the gas components in real time (Figure 1B). The result shows that the gas can be considered pure (impurity <10 ppm) after purging at a rate of 0.4 mL/min for 2 h (Figure S1), which is consistent with the predicted time (see the calculation in method details).

To check the feasibility of Li-CO_2_ batteries, different cathodes were tested at an area current density of 17.7 μA cm^−2^ (Figure 1C). The discharge voltages of these cathode materials are different which is caused by their inherent catalytic activities. It is clear that the discharge voltages of super P, carbon nanotube (CNT), graphene, and nanoporous Au (NPG, Figure S2) drop to below 2.0 V quickly, which is much lower than the claimed ∼2.7 V in other works with much higher current densities.^4^^,^^5^^,^^32^^,^^35^^,^^36^^,^^37^^,^^38^^,^^39^^,^^40^^,^^41^ Although the batteries with Ru/CNT and Pd/CNT cathodes have higher capacities above 2.0 V, the voltages are much lower than 2.5 V. For the batteries with super P, CNT, graphene, and NPG cathodes, only small capacities can be delivered above 2.0 V at such a small current density, while these cathode materials exhibit good performance in previous reports.^4^^,^^5^^,^^35^^,^^42^ When introducing 2% O_2_ into the CO_2_ environment, the discharge voltages of all the batteries slightly increase (Figure 1D). This indicates that O_2_ has been involved in the discharge reactions to lift the discharge voltage, but the influence is limited because the small amount of O_2_ could be consumed quickly. Further increasing the amount of O_2_ in CO_2_ to 5% and 10% makes most of the discharge profiles (except NPG) similar to those of Li-O_2_ batteries with higher discharge plateaus (Figures 1E, 1F, and S3A). When O_2_ is involved, it is first reduced to form O_2_^−^, followed by integration with CO_2_, and finally Li_2_CO_3_ forms. In the whole reduction reaction, only O_2_ is reduced, so the observed discharge voltage is actually contributed by O_2_ reduction. Therefore, the O_2_-involved Li-CO_2_ batteries display similar performance with Li-O_2_ batteries.^3^ Naturally, higher concentration of O_2_ facilitates the reduction kinetics and improves the discharge voltage. The discharge performance of these batteries at a higher current density of 44.2 μA cm^−2^ is demonstrated in Figures S3B−S3E. Despite with a similar trend in capacity and voltage change with higher O_2_ concentration, the high current density lowers the discharge plateaus and capacities. The comparison of the discharge capacities of Li-(O_2_/)CO_2_ batteries at different conditions is illustrated in Figure 1G, where we can conclude that higher content of O_2_ and lower discharge current density can lead to a higher capacity. However, generally, the high concentration of CO_2_ enables sluggish reaction kinetics. In addition, other cathode materials including g-C_3_N_4_, CNT sponge, Ir/C, MoSe_2_, CoS, Co_0.5_Fe_0.33_WO_4_, and MoS_2_ also display low discharge plateaus in Li-CO_2_ batteries (Figure S4).

**Figure 2:**
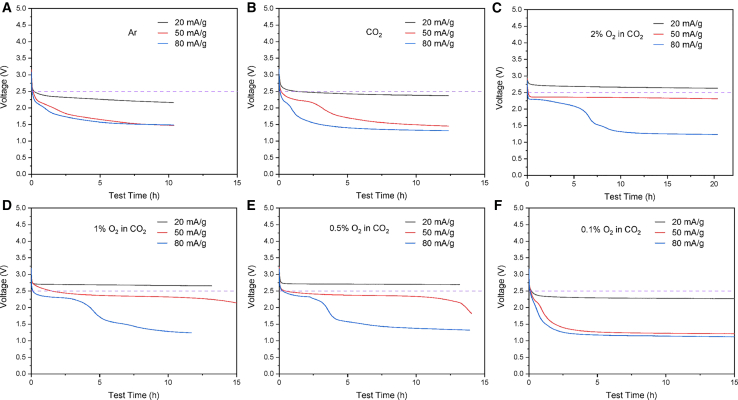
The lower limit of O_2_ that can change the discharge voltage of Li-CO_2_ batteries with CNT cathodes Discharge voltage of Li-CNT batteries in the (A) Ar, (B) CO_2_, (C) 2% O_2_/CO_2_, (D) 1% O_2_/CO_2_, (E) 0.5% O_2_/CO_2_, and (F) 0.1% O_2_/CO_2_. The current densities of 20, 50, and 80 mA/g represent 5 μA, 12.5 μA, and 20 μA, respectively.

To check the lower limit of O_2_ concentration that could significantly change the voltage of Li-CO_2_ batteries, we decreased the current density to 20, 50, and 80 mA/g, which corresponded to 5, 12.5, and 20 μA, and lowered the O_2_ concentration to 1%, 0.5%, and 0.1%. When we compare the discharge voltages of Li-CNT batteries in the Ar and pure CO_2_ (Figures 2A and 2B), it is found that a low current density of 20 mA/g can enable the batteries with a high discharge plateau (2.3–2.4 V), while higher rates induce fast voltage drop. When 2% O_2_ is introduced, the discharge voltages improve obviously and a plateau at 2.7 V is observed at 20 mA/g (Figure 2C). The results are much better than those in Figure 1D, indicating lower rates can lift the voltage. Then, we lowered the O_2_ concentration to 1% and 0.5% (Figures 2D and 2E). The results are similar to the situation with 2% O_2_ in Figure 2C. However, when 0.1% O_2_/CO_2_ was used, the voltage cannot recover to above 2.5 V, even at 20 mA/g. Therefore, we have identified a trace amount of O_2_, as low as 0.5% O_2_ in CO_2_, could change the battery performance dramatically. In an imperfectly sealed battery testing system, such a low O_2_ concentration is easily achieved.

The performance of Li-CO_2_ batteries with different electrolytes (1 M LiTFSI/TEGDME, 1 M LiFSI/DMSO, and 0.1 M LiTFSI/[IL-DMSO]) was also checked (Figure S5A). We can see that the discharge plateaus vary in these electrolytes due to their different viscosity, CO_2_ solubility, etc., but they are all lower than 2.5 V at a low current density of 17.7 μA cm^−2^. Furthermore, the cycling performance of Li-CO_2_ batteries with three kinds of redox mediators (RMs: iron phthalocyanine, benquine [BQ], and LiBr) were checked (Figure S5C). Due to the different redox activities and potentials of these RM molecules, the discharge potentials of the Li-CO_2_ batteries also varied. Compared with the cycling curve of the Li-CO_2_ battery without RMs (Figure S5B), no performance improvement can be observed with the introduction of RMs. The voltage plateaus of the battery with BQ should be ascribed to the redox reactions of BQ (Figure S6). Considering these RMs have been widely used in Li-O_2_ batteries due to their suitable redox potentials and previously reported Li-CO_2_ batteries have shown similar discharge voltages with Li-O_2_ batteries, they should be also effective in Li-CO_2_ batteries. The negligible effect of these RMs shown here indicates that the actual reduction potential of CO_2_ in Li-CO_2_ batteries is much lower than the redox potentials of these RMs.

**Figure 3:**
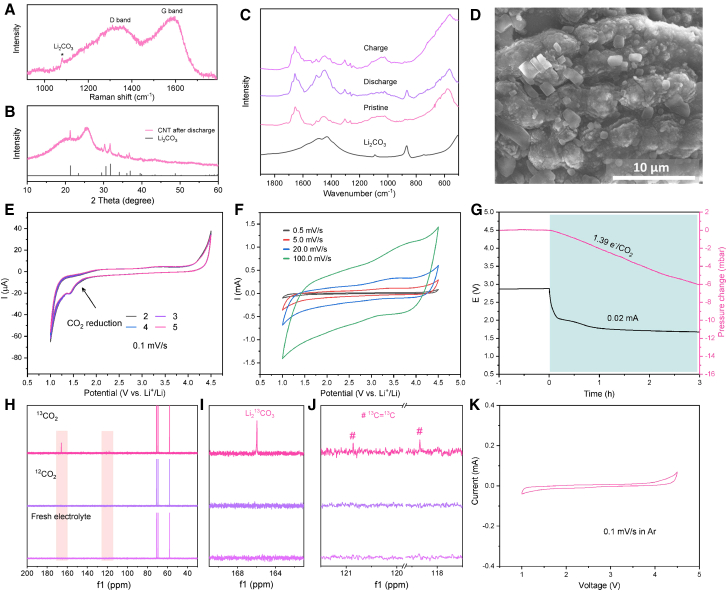
Evidences of viable Li-CO_2_ batteries at lower discharge voltage (A) Raman spectrum of NPG cathode after discharge in Li-CO_2_ battery. (B) XRD patterns of CNT after discharge in Li-CO_2_ battery. (C) FTIR spectra of CNT cathodes after discharge and charge. (D) Discharge product on NPG cathode. (E and F) CV curves of Li-CO_2_ batteries with CNT cathodes at 0.1 mV/s and higher scan rates between 1.0 and 4.5 V. (G) CO_2_ consumption during discharge in Li-CO_2_ battery at 0.02 mA. (H–J) ^13^C NMR of the discharge products on NPG. (K) CV curve of Li-CNT battery in the Ar.

Besides O_2_, other trace amount of gases (N_2_, H_2_O, and H_2_) in electrolyte, or feeding gas cannot influence the discharge behavior much or lift the discharge voltage to above 2.5 V. As to the influence of N_2_, it is reported that the discharge voltage of Li-N_2_ battery is ∼1 V,^43^ much lower than Li-CO_2_ batteries in this work. This means CO_2_ is first to be reduced compared with N_2_., thus the N_2_ influence can be excluded. When H_2_O is involved, this reaction CO_2_ + 2H_2_O + 4Li^+^ + 4e^−^ → C + 4LiOH, could proceed to generate LiOH.^18^ However, no LiOH can be detected in previous Li-CO_2_ battery reports^44^^,^^45^ and this work (Figures 3A–3C). In addition, the concentration of H_2_O in air is much lower than O_2_ and N_2_, subtle H_2_O involvement could be consumed instantly thus cannot change the Li-CO_2_ battery evidently. Moreover, the discharge potential of the above reaction is 2.26 V,^18^ which is lower than the 2.5 V reported by previous works. Based on these evidences, we believe the trace amounts of gases such as N_2_ and H_2_O have very limited impact on Li-CO_2_ batteries. To check the possible influence of adsorbed gases, we tested Li-based batteries with different catalysts in Ar (Figure S3F) and found that none of these batteries could discharge above 2.5 V, but the cathode could deliver some capacity at low voltages due to Li^+^ adsorption or intercalation at the cathode. Besides, considering the low content of possible gas adsorption, there would be a short plateau during discharge caused by gas participation. Again, we cannot see any possible reaction caused by adsorbed gas from the discharge curve.

The above results confirm that the discharge performance of Li-CO_2_ batteries has been overestimated for a long time, and higher temperature and RM addition only have limited effect for improving the battery performance. However, the performance improvements with increased O_2_ concentration in CO_2_ have validated the critical role of O_2_ in Li-CO_2_ batteries. The influence of O_2_ and the lower discharge voltage of Li-CO_2_ battery can also be proved by the gas switch test and cyclic voltammetry (CV) results (Figure S7). After discharging in CO_2_ for 1.8 h, O_2_ was purged to replace CO_2_. This simple gas exchange makes the discharge voltage recover to a high value for CNT and Pd/CNT cathodes and lasts for a long time. Once oxygen is purged, it can be firstly reduced to O_2_^−^ prior to CO_2_ due to its favorable reaction kinetic and thermodynamically high reduction potential than CO_2_. Therefore, the higher reduction potential of O_2_ enables the sharp increase of discharge voltage. However, the increased voltages only sustain for several minutes for Ru/CNT, super P, and graphene, and there is even no voltage change for NPG (Figure S7A) that may be resulted by surface passivation. The voltage increase can also be observed in Li-CO_2_ batteries with MoS_2_ as the cathodes (Figure S7B). Especially, in the Li-O_2_ battery with CNT cathode (Figure S7C), when O_2_ is replaced by CO_2_, the voltage slumps from 2.7 V to 1.2 V, and when the O_2_ is refilled into the CO_2_ to form a mixture of O_2_/CO_2_ (v/v, 1:1), the voltage recovers to above 2.5 V. The slump can be repeated after CO_2_ is repurged into the testing system. It is noted that there are no reduction peaks above 2.0 V in the CV curves of the Li-CO_2_ battery, implying CO_2_ is not active at this stage (Figure S7D). In contrast, the batteries with O_2_ involvement have obvious reduction peaks at 2.4 V and higher O_2_ concentrations lead to larger peak currents.

### The feasibility of Li-CO_2_ batteries

Above analysis has proved that the unintentionally involved O_2_ can greatly improve the performance of Li-CO_2_ batteries, but this has been long neglected in the previous Li-CO_2_ works because of the flawed experiment design. However, the feasibility of true Li-CO_2_ batteries has not been discussed yet. Thermodynamically, the discharge process of Li-CO_2_ batteries is spontaneous because the Gibbs free energy change of reaction 4Li + 3CO_2_ → 2Li_2_CO_3_ + C is −1080 kJ/mol with a corresponding E^o^ = 2.80 V vs. Li^+^/Li.^46^ However, there were still some researchers doubting the feasibility of Li-CO_2_ batteries since no observed redox reactions in their studies. This claim is obviously inconsistent with the theoretical result. Nevertheless, we cannot explain why only negligible capacities could be delivered for some Li-CO_2_ batteries so far. In the above experiments, we found some hidden information, which may help resolve this problem. Considering the gas switch from CO_2_ to O_2_ in Figure S7A cannot make the voltages recover for a long time (limited capacity) for the batteries with NPG, Ru/CNT, Super P, and graphene cathodes, we guess a passivation layer has formed on these cathodes that block the reaction sites for further proceeding the discharge reaction, and the passivation may come from the discharge products of Li-CO_2_ batteries.

To investigate this abnormal phenomenon, a series of experiments were conducted. After being discharged to 1.0 V in pure CO_2_ environment, the NPG cathode was characterized by Raman spectrometry to check whether there were any deposited discharge products. Beyond our expectation, the peaks from Li_2_CO_3_ (1,080 cm^−1^) and carbon (D band at 1,350 cm^−1^ and G band at 1,590 cm^−1^) can be clearly observed (Figure 3A). The carbon-free gold avoids the interference of carbon from cathode, thus the above carbon signal should be attributed to the formed carbon after discharge. Further, for the discharged CNT cathode, there are strong Li_2_CO_3_ peaks at 21.3°, 30.6°, and 31.8° in the XRD patterns (Figures 3B and S8). The emergence and extinction of Li_2_CO_3_ peaks at 878, 1432, and 1488 cm^−1^ in the FTIR spectra also confirms the formation and decomposition of Li_2_CO_3_ after discharge and charge for 0.4 mAh (Figure 3C). Besides, the formation of Li_2_CO_3_ on NPG can be directly visualized by SEM (Figure 3D), and the Li_2_CO_3_ piles on CNT can be seen in Figures S9A and S9B. These results confirm Li_2_CO_3_ is the discharge product for Li-CO_2_ batteries and will cover the active cathode surface during the discharge process (Figure S9D). SEM images of CNT cathodes at different discharge and charge stages verify that the Li_2_CO_3_ is rechargeable (Figure S10). Therefore, a rechargeable Li-CO_2_ battery with Li_2_CO_3_ and C as the discharge products is feasible. In addition, the discharge product morphologies and XRD patterns of graphene, Ru/CNT, and super P have been shown in Figure S11. These results demonstrate that a product layer on the catalysts and XRD patterns confirm the layer is Li_2_CO_3_. Besides, some reports using RuO_2_/CNT^19^ and Pt/SS^47^ also confirm the formation of Li_2_CO_3_.

From the CV curves in Figure 3E, we can see that the CO_2_ fixation peak appears around 1.5 V at a slow scan rate of 0.1 mV s^−1^, which is close to the discharge plateau in Figure 1C, while this peak cannot be discerned at higher scan rates of 0.5 mV/s, 5.0 mV/s, 20 mV/s, and 100 mV/s (Figure 3F). Faster sweep rates result in increased CV area, and the CV curves of the Li-CO_2_ battery display rectangle-like shapes, which are typical capacitive behavior without redox reactions. In this case, the slow kinetics of the Li-CO_2_ battery are overlapped by the capacitive current, which is the reason why some works using high sweep rates claim the Li-CO_2_ battery is not viable.^21^^,^^24^ To further reveal the electrochemical reaction that happened in the Li-CO_2_ battery, operando pressure monitor system was used to check the electron transfer per CO_2_ during the discharge process. A value of 1.39 e^−^/CO_2_ is obtained (Figure 3G), which is consistent with the theoretical value of 1.33 e^−^/CO_2_ for the reaction of 4Li + 3CO_2_ → 2Li_2_CO_3_ + C. This also directly confirms that CO_2_ is electrochemically consumed during discharge. Despite a small current of 0.02 mA (17.7 μA/cm^2^) has been used to minimize the capacitance here, it still cannot be completely removed, thus the experimental e^−^/CO_2_ value, 1.39, is a little bit higher than the theoretical one. In addition, isotope labeling was used to check the discharge products of Li-^13^CO_2_ battery with nanoporous Au cathode. After discharge, the cathode and PP membrane were immersed in D_2_O, and the solution was detected by ^13^C NMR. As indicated in Figures 3H–3J, clear signals of Li_2_^13^CO_3_ at 165.9 ppm and ^13^C = ^13^C at 120.9 and 118.3 ppm can be observed. In addition, Raman spectroscopy of pristine NPG (Figure S2B) does not show D and G bands, suggesting there is no extraneous carbon on the NPG. Therefore, the carbon observed after discharge should be attributed to CO_2_ reduction. To the best of our knowledge, this is the first verification of Li_2_CO_3_ and C using isotope labeling and NMR in Li-CO_2_ batteries. We also note that the electrolyte during discharge is stable, as confirmed by CV curve in Figure 3K. In Figure S12, the ^1^H NMR spectra of the electrolytes after discharge are identical without new emerged peaks, confirming no electrolyte decomposition. Besides, CO is not observed during discharging the battery in the DEMS test (Figure S13).

### Theoretical calculation

**Figure 4:**
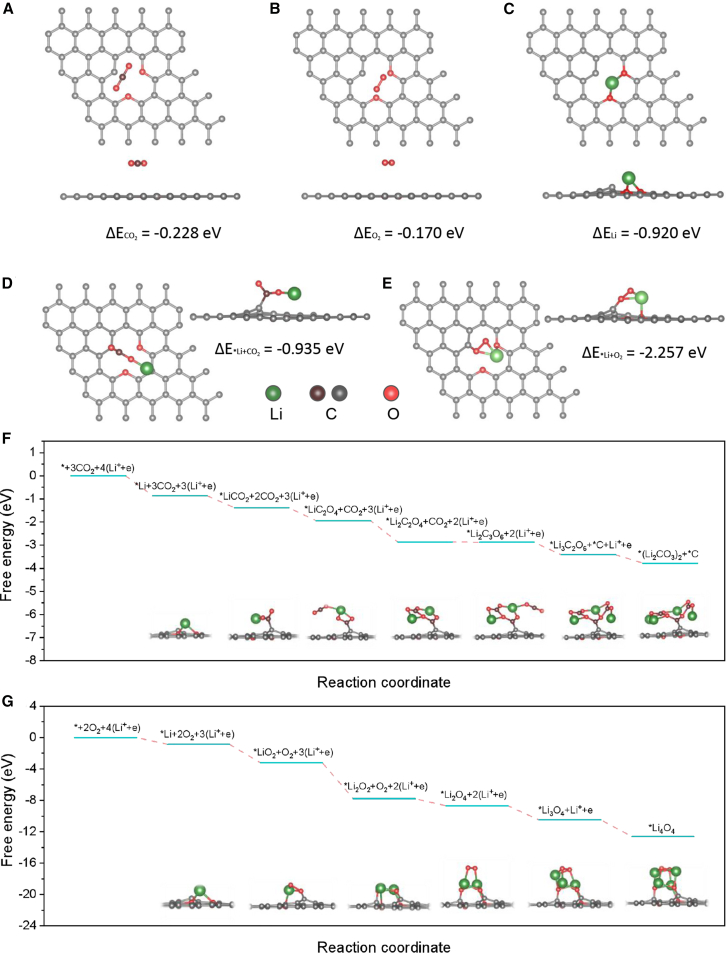
Computational study of the reaction kinetics of Li-CO_2_ battery and Li-O_2_ battery (A–E) Top and side views of the optimized energetically most favorable structure of (A) ∗CO_2_, (B) ∗O_2_, (C) ∗Li, (D) ∗Li+CO_2_, and (E) ∗Li+O_2_ absorbed on defected carbon surface. The green ball is Li atom and the red one is oxygen atom. The brown ball and gray ball are carbon atoms on CO_2_ and CNT, respectively. (F and G) Schematic Gibbs free energy diagrams of the formation of Li_2_CO_3_ and C (F), and Li_2_O_2_ (G) during the discharge process.

The reaction mechanisms of Li-CO_2_ and Li-O_2_ batteries were then studied by performing density functional theory (DFT) calculations to obtain the growth pathways of Li_2_CO_3_ and Li_2_O_2_. For each step of the elementary reaction, all possible configurations were calculated, and the one with the lowest energy was chose (Figure 4). The CNT surface model (∗ denotes the CNT substrate, Figures 4 and S14) was built with a defected C atom and two doped O atoms because the CNT used in the experiment was not flawless (Figure 4A).^48^ According to our calculations (Figures 4A–4C), the absorption energy of CO_2_ (−0.228 eV) or O_2_ (−0.170 eV) on the CNT is smaller than that of Li+ (−0.920 eV). Therefore, at the beginning of the discharge process of Li-CO_2_ and Li-O_2_ batteries, Li^+^ is the most possible species absorbed on the CNT surface. After Li^+^ is absorbed on the CNT, O_2_ is more easily reacting with ∗Li due to the higher absorption energy of ΔE_O2+∗Li_ = −2.257 eV (Figure 4E) than that of ΔE_CO2+∗Li_ = −0.935 eV (Figure 4D). Meanwhile, the charge density difference also obviously demonstrates that the interaction strengths between ∗ and Li, as well as ∗Li and CO_2_ or O_2_ (Figure S14). There is no charge density between ∗ and CO_2_ or O_2_ (Figures S14A and S14B), which verifies their weak interactions, being consistent with their low absorption energies in Figures 4A and 4B. On the contrary, there is a strong charge transfer process between ∗ and Li^+^ (Figure S14C), ∗Li and O_2_ (Figure S14D), and ∗Li and CO_2_ (Figure S14E).

To further prove the slower kinetic process of Li-CO_2_ batteries, the possible nucleation paths with reaction intermediates for Li-CO_2_ and Li-O_2_ batteries are shown in Figures 4F and 4G. The optimized discharge reaction paths of Li-CO_2_ batteries proceed as follows:

(Reaction 1) Li^+^ + e^−^ + ∗ → ∗Li

(Reaction 2) ∗Li + CO_2_ → ∗LiCO_2_

(Reaction 3) ∗LiCO_2_ + CO_2_ → ∗LiC_2_O_4_

(Reaction 4) ∗LiC_2_O_4_ + Li^+^ + e^−^ → ∗Li_2_C_2_O_4_

(Reaction 5) ∗Li_2_C_2_O_4_ + CO_2_ → ∗Li_2_C_3_O_6_

(Reaction 6) ∗Li_2_C_3_O_6_ + Li^+^ + e^−^ → ∗Li_3_C_3_O_6_ + ∗C

(Reaction 7) ∗Li_3_C_2_O_6_ + Li^+^ + e^−^ → ∗(Li_2_CO_3_)_2_

In our calculations, the rate-determining step (RDS) of (Li_2_CO_3_)_2_ nucleation process is Reaction 5, whose free energy is only −0.009 eV, while the RDS of (Li_4_O_4_) is the reaction of Li^+^ + e^−^ + ∗ → ∗Li with a free energy of −0.865 eV. The RDS’s difference makes the discharge reaction of Li-CO_2_ batteries kinetically slower than that of Li-O_2_ batteries. Moreover, the total free energy change of the nucleation process for (Li_2_CO_3_)_2_ is only −3.779 eV (Figure 4F), which is far less than the −12.609 eV for Li_4_O_4_ (Figure 4G), thus it is clear that, in terms of the discharge processes of the Li-CO_2_ battery and Li-O_2_ battery, Li_2_O_2_ is more easily formed than Li_2_CO_3_ thermodynamically. We present another two discharge paths in Figure S15 that shows similar results. The formation of Li_2_O_2_ after discharge in Li-O_2_ battery has been proved in Figure S16. These calculation results obviously demonstrate that the kinetics and thermodynamics of Li-O_2_ batteries are superior to those of Li-CO_2_ batteries. Therefore, at the same current density, the discharge voltage of Li-CO_2_ battery is much lower than that of Li-O_2_ battery.

### Equilibrium potentials

Up to now, we can conclude that O_2_ permeation is responsible for the high discharge voltage of the previously reported, so-called Li-CO_2_ batteries. However, a true Li-CO_2_ battery is feasible through the reaction of 4Li + 3CO_2_ → 2Li_2_CO_3_ + C, despite that this reaction happens at a lower voltage (<2.5 V) and is kinetically sluggish. Those who doubted the feasibility of Li-CO_2_ batteries actually neglected the reactions that happened below 2.0 V.

**Figure 5:**
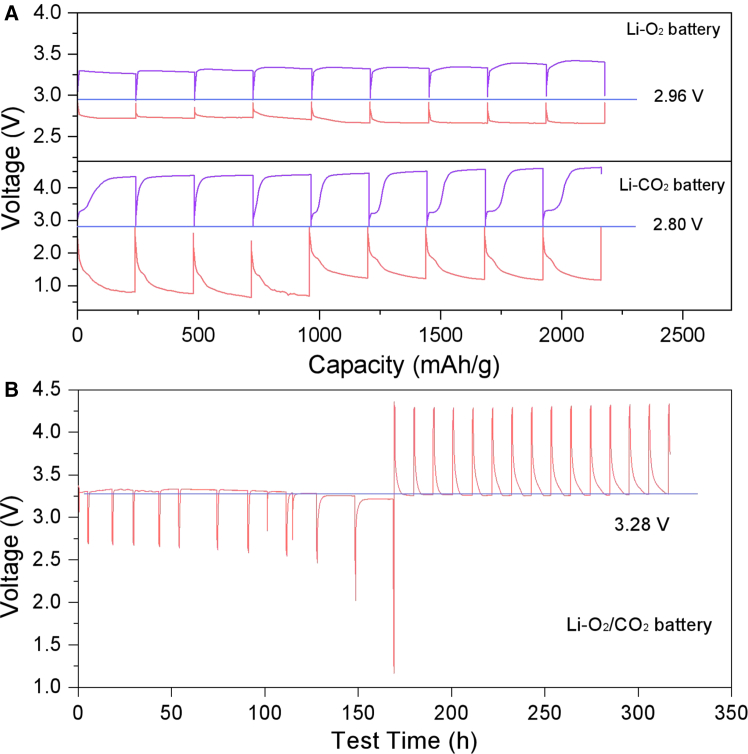
Determination of the equilibrium potentials of Li-O_2_ and Li-CO_2_ batteries (A and B) GITT tests of the (A) Li-O_2_ battery, Li-CO_2_ battery and (B) Li-O_2_/CO_2_ (1:1) battery that being discharged or charged for 0.5 h at 0.05 mA. The resting time are 3 h, 5 h and 10−20 h for the Li-O_2_ battery, Li-CO_2_ battery, and Li-O_2_/CO_2_ battery, respectively.

Galvanostatic intermittent titration technique (GITT) can determine the equilibrium potential (E_eq_) of a battery by measuring its transient and steady states. In view of our unique battery testing system, GITT was used to test the equilibrium potentials of Li-O_2_, Li-CO_2_, and Li-O_2_/CO_2_ (1:1) batteries at 0.05 mA with 0.5 h for discharge or charge (Figure 5). The relaxation periods for the batteries were tailored in advance. The E_eq_ of the Li-O_2_ battery is 2.96 V (Figure 5A), which is consistent with the theoretical value and accordingly proves the reliability of our rigorous experimental setup. The Li-O_2_ battery displays a low charge voltage of 3.3 V and a high discharge voltage of 2.7 V. In a 3-h rest, the voltage could reach the equilibrium state. For the Li-CO_2_ battery, the E_eq_ is 2.80 V (Figure 5A), the same value to the calculated result mentioned above. Many works related to Li-CO_2_ batteries have failed to report this potential by GITT correctly, which may be influenced by the involved O_2_. However, the discharge voltage degrades to <1.0 V quickly when current (0.05 mA) is applied. An overpotential of ∼1.8 V is observed during discharge, and the charge voltage is approaching 4.5 V, thus more time (5 h) is needed to reach a steady state. Being different from the above two equilibrium values, the E_eq_ of Li-O_2_/CO_2_ (molar ratio 1:1) battery is 3.28 V (Figure 5B), and the required time for a steady state is further prolonged (10–20 h). Therefore, the value of E_eq_ can be considered as a signal of gas purity in the battery system. Once O_2_ is involved in Li-CO_2_ batteries, the equilibrium potential would increase. We can also conclude that the formation of Li_2_CO_3_ leads to a longer rest time to reach the equilibrium states, which may be resulted by the low Li^+^ diffusion rate in Li_2_CO_3_. Furthermore, the discharge voltage of the Li-CO_2_ battery is much lower than the Li-O_2_ battery, again confirming the slow kinetics of Li-CO_2_ batteries.

### Sealibility check

Through the experimental and theoretical investigation, we have confirmed that unconscious O_2_ in Li-CO_2_ batteries could greatly improve the performance, and the actual discharge reaction of Li-CO_2_ batteries is sluggish. In the context of reducing carbon emissions globally, Li-CO_2_ batteries have their values to study for CO_2_ fixation and transformation. However, the research condition must be rigorous to ensure true battery performance and result reproducibility. In future Li-CO_2_ works, to avoid the influence of O_2_ in Li-CO_2_ batteries, measures must be taken to detect the purity of the gas environment and remove the permeant O_2_ before battery cycling.

**Figure 6:**
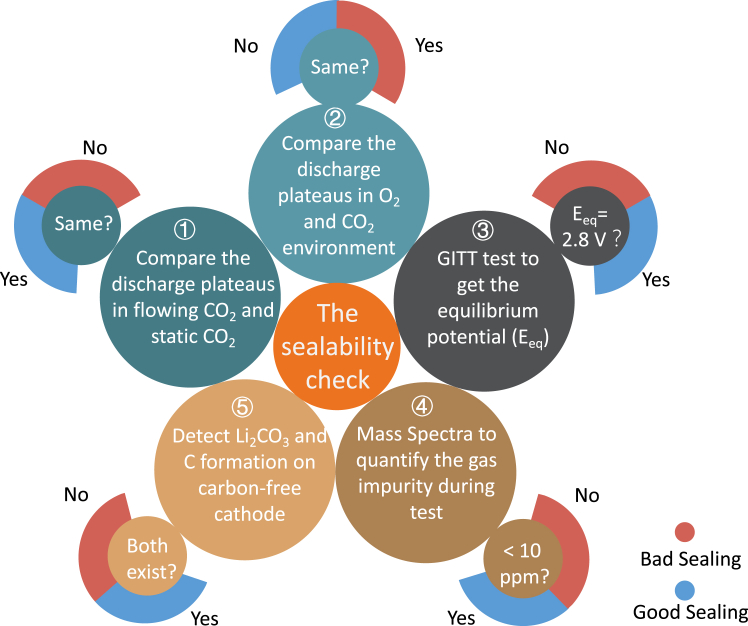
Strategies to check the sealability of Li-CO_2_ battery testing system Five ways with evaluation methods are listed in the diagram. The “Yes” or “No” is the judgment result of the questions in the small circles and the color of adjacent circular arc indicates the corresponding sealing condition of the testing system. By the results of the experiments, one can estimate the sealing of the testing system.

Here, based on the results from this work, some instructions are proposed to give hints whether O_2_ participates in the discharge reactions of Li-CO_2_ batteries (Figure 6):

(1) Compare the discharge voltage of Li-CO_2_ batteries with flowing gas and static gas. Continuous CO_2_ blowing can prevent oxygen permeation from the air. If their discharge voltages are the same, a pure CO_2_ testing system is confirmed, because no oxygen is involved in the static gas. Otherwise, the higher discharge voltage induced by the O_2_ permeation may remind the users to consider the leakage of the environment.
(2) Compare the discharge plateaus of Li-CO_2_ battery with Li-O_2_ or Li-O_2_/CO_2_ batteries. We have confirmed the discharge voltage of Li-CO_2_ batteries is lower than Li-O_2_ batteries experimentally in Figures 5A, S3, S7A–S7C, and theoretically in Figures 4F and 4G. Even though some catalysts may have good catalytic effects, there remain voltage differences under varied gas components. Gas switching during discharge is more direct (Figure S7C). When purposely introducing O_2_ in the Li-CO_2_ batteries during discharge, if no voltage change can be observed, this means O_2_ already exists in the so-called Li-CO_2_ batteries, thus the sealibility of the testing system should be carefully checked.
(3) We have demonstrated that the GITT curves of Li-O_2_, Li-CO_2_, and Li-O_2_/CO_2_ batteries give their different equilibrium potentials due to the varied gas environments (Figure 5). Therefore, GITT can be considered as a solution to identify the true gases in the testing system. If the equilibrium voltages of Li-CO_2_ batteries from GITT test are not ∼2.8 V, some contaminant O_2_ may be involved in the poor-sealed system during the test.
(4) The gas components can be directly detected by a mass spectrometer (MS). Hence, connecting the battery system to an MS with high-purity (contaminant gas <10 ppm, 5N) Ar or CO_2_ can help check the sealibility. If the system is well sealed, the signals of O_2_ or N_2_ will be very low because of the high purity of the carrier gas. If the result shows a high content of O_2_ or N_2_, the sealibility should be improved. Therefore, this method can give unambiguous result to judge the sealibility of the testing environment.
(5) The formation of Li_2_CO_3_ and C during discharge of Li-CO_2_ batteries has been verified using NPG as the cathodes in this work. In Li-O_2_/CO_2_ batteries, the discharge product is solely Li_2_CO_3_. The difference between Li-CO_2_ and Li-O_2_/CO_2_ batteries is the existence of carbon after discharge. To avoid the carbon source from cathode, a carbon-free cathode should be adopted to check whether the two products can be simultaneously formed in Li-CO_2_ batteries after discharge. The formation of C can be as a signal of good tightness.

The first three ways directly adopt electrochemical test, the fourth one utilizes characterization equipment, while the fifth one combines electrochemical and Raman tests. In practice, one or more of these strategies can be taken to ensure rigorousness. Despite the importance of the sealing status of the testing systems, they have long been ignored in the previous works, as summarized in Table S1. With the above strategies, we can easily confirm whether the Li-CO_2_ battery is a true one or not.

In summary, we have developed a rigorous battery testing system to reveal the true performance of Li-CO_2_ batteries. It is found that the high performance of the previously reported Li-CO_2_ batteries cannot be reproduced and can be only achieved with the help of unconscious involved O_2_. Despite the performance of true Li-CO_2_ batteries above 2.0 V is poor, and the discharge plateau is much lower than that of Li-O_2_ batteries due to the sluggish kinetics of CO_2_ reduction, the Li-CO_2_ battery is indeed feasible. Also, we have explored the reactions that happened below 2.0 V and confirmed the discharge reaction of Li-CO_2_ batteries is 4Li + 3CO_2_ → 2Li_2_CO_3_ + C through systematic characterizations, which can definitely explain why some of previous works deny the electro-activity of CO_2_ in Li-CO_2_ batteries with a cutoff discharge voltage above 2.0 V. Finally, five methods to check the sealability of Li-CO_2_ batteries have been proposed as a reference for future works. All in all, the long-lasting controversy on the feasibility of Li-CO_2_ batteries has been resolved. By clarifying this issue, we hope more works can be inspired to study the mechanisms of this battery system.

### Limitations of the study

Our testing system suffers from severe electrolyte volatilization due to continuous gas flow, which could affect long-term cycling stability (though this was not the focus of the study). The DFT calculations used a simplified CNT model with defects and oxygen doping, which may not fully represent all real-world cathode materials.

## Resource availability

### Lead contact

Further information and requests for resources and reagents should be directed to and will be fulfilled by the lead contact, Gang Huang (ghuang@ciac.ac.cn).

### Materials availability

This study did not generate new unique reagents.

### Data and code availability

• Data reported in this article will be shared by the lead contact upon request.
• This study does not report the original code.
• Further details and data supporting the findings of this study can be obtained from the lead contact upon reasonable request.

## Acknowledgments

The authors thank the supports from the 10.13039/501100012166National Key R&D Program of China (2021YFF0500600), 10.13039/501100001809National Natural Science Foundation of China (U22A20437, 52171194, 52271140, and 22209138), Strategic Priority Research Program of the Chinese Academy of Sciences (XDB1040101), 10.13039/501100002858China Postdoctoral Science Foundation (2024M750272), Key Deployment Program of the Chinese Academy of Sciences (KGFZD-145-23-54) and National Natural Science Foundation of China Outstanding Youth Science Foundation of China (Overseas).

## Author contributions

K.C. and G.H. conceived the idea for solving the controversy of Li-CO_2_ batteries. K.C. designed the rigorous testing system, J.-Y.D. and K.L. conducted the theoretical calculation. K.C., H.Z., Y.F., D.-Y.Y., G.H., and J.W. designed and conducted the experiments. All authors participated in the result analysis. K.C., J.-Y.D., and G.H. wrote the manuscript and all authors revised it.

## Declaration of interests

The authors declare no competing interests.

## STAR★Methods

### Key resources table

**Table:** 

REAGENT or RESOURCE	SOURCE	IDENTIFIER
**Other**		
Lithium trifluoromethanesulfonate (LiCF_3_SO_3_)	Aladdin	L101046
Lithium bis(fluorosulfonyl)imide (LiFSI),	Aladdin	L157764
Lithium bis-trifluoromethane sulfonimide (LiTFSI)	Aladdin	B102576
Molybdenum disulfide (MoS_2_)	Aladdin	M104967
Tetraethylene glycol dimethyl ether (TEGDME)	Aladdin	T111151
Polyvinylidene fluoride (PVDF) powder,	Sigma-Aldrich	#182702
Ion liquid (1-ethyl-3-methylimidazolium chloride)	Sigma-Aldrich	#53096
Dicyandiamide	Sigma-Aldrich	D76609
Potassium chloropalladite (K_2_PdCl_4_)	Sigma-Aldrich	#205796
ruthenium(III) chloride hydrate (RuCl_3_·H_2_O)	Sigma-Aldrich	#119247
Superdry acetonitrile (CH_3_CN)	J&K Scientific	#63819
Dimethyl sulfoxide (DMSO)	J&K Scientific	#70710
N-methyl-2-pyrrolidone (NMP)	J&K Scientific	#4325736
The standard gas (0.1%, 0.5%, 1%, 2%, 5%, 10% O_2_ in CO_2_)	Zhonghaoguangming Co. Ltd., Dalian	Customized

### Method details

#### Electrolyte

The electrolyte was 1 M LiCF_3_SO_3_/TEGDME except being mentioned specifically. The water content in the electrolyte was less than 30 ppm determined by 831 KF Coulometer, Metrohm, Switzerland. The volume ratio of IL and DMSO in the 0.1 M LiTFSI/(IL:DMSO) electrolyte was 1:3.

#### Preparation of cathode materials

CNT cathode: CNT and PVDF were mixed at a mass ratio of 9:1 in NMP (N-methyl-2-pyrrolidone). After grinding for 30 minutes, the slurry was sprayed on carbon paper. Then the paper was vacuum dried overnight at 80°C to remove NMP. Finally, the paper was cut into round pieces and stored in glove box which was filled by Ar (O_2_, H_2_O<0.1 ppm). The CNT loading was 0.1 mg/piece.

Ru/CNT cathode: firstly, 1 g F127 was dissolved in 300 ml deionized water followed by the addition of 0.6 g CNT. After stirring the mixture for 24 hours, a 30 ml aqueous solution containing 0.6 g RuCl_3_·H_2_O was added. The obtained mixture was further stirred for 24 hours and then dried by vaporizing water to get solid product. Finally, the product was transferred to tube furnace and heated at 300°C for 3 hours with a ramping rate of 5°C/min under 5% H_2_/Ar flowing gas. The active material mass loading was 0.1 mg/piece.

Pd/CNT cathode: 30 mg CNT was first added to 50 ml ethylene glycol followed by sonication. Then, 2 ml K_2_PdCl_4_ aqueous solution with a Pd content of 6 mg/ml was added to the mixture. After stirring 30 min, the mixture was heated to 130°C and the temperature was held for 2 h. Finally, the Pd/CNT could be collected after repeated centrifugal process and washing. The cathode pieces can be obtained by following the procedures mentioned in CNT cathode. The Pd/CNT loading was 0.1 mg/piece.

Pt/C cathode: 45 mg Pt/C (Pt wt% = 10%) and 5 mg PVDF were mixed and milled in NMP. The slurry was dropped on carbon paper (12 mm in diameter) with a mass loading of 2.8 mg or 0.19 mg.

Graphene cathode: The procedure was the same as that of CNT cathode. The mass loading was 0.1 mg/piece.

MoS_2_ cathode: 1.6 g MoS_2_ and 0.4 g graphite were dispersed in 5 ml ethanol followed by ball milling for 6 h (600 r/min for 30 min, and then rest 10 min). Then the slurry was dried at 70°C overnight. The prepared material, Super P and PTFE were mixed at a mass ratio of 7:2:1. The mixture was pressed to thin pieces and used as cathodes directly after drying. The MoS_2_ loading was 0.1 mg/piece.

Synthesis of g-C_3_N_4_: pristine multi-walled carbon nanotubes (CNT, Sigma-Aldrich) were sonicated in a 3:1 v/v solution of sulfuric acid (98%) and nitric acid (70%) at room temperature for 24 h to introduce hydrophilic functional groups on the surface. After repeatedly washing, the treated CNT was stirred in a 5 M nitric acid solution for 24 h to eliminate the metal impurities. After this, 25 mg of CNT was dispersed in 100 mL water, and then 250 mg (∼6 mmol) of dicyandiamide (DCDA) was added and stirred overnight at 50°C. Note here that the amount of DCDA was doubled to ensure coordination efficiency and account for melamine sublimation during the polymerisation of g-C_3_N_4_ from DCDA. Finally, the mixture was lyophilized and annealed at 600°C in N_2_. The loading was ∼0.3 mg after electrode preparation procedures similar to the CNT electrode.

Nanoporous gold (NPG) cathode: a gold-silver (Au-Ag) alloy film was firstly cut into round pieces (12 mm in diameter). Then the pieces were de-alloyed by HNO_3_ to remove the Ag. After washing in ethanol, nanoporous gold pieces were dried in a bake oven at 80°C.

#### Battery assembly and electrochemical tests

All the batteries were tested in ECC-AIR cells (EL-cell GmbH, Germany) to ensure gas tight during the test. The structure of ECC-AIR cell and the assembly procedures could be detailedly seen in our previous report.^3^ After assembly in glove box (filled by Ar and the content of H_2_O and O_2_ were less than 0.1 ppm), the batteries were transferred outside to connect with flowmeters so that constant gas could pass through the batteries.

The batteries were rested at least 3 h to ensure gas purity and electrolyte wetting. Battery cycling tests and other electrochemical tests were conducted on LAND (CT2100A) (LANHE, China) multi-channel battery testing system and VMP-300 electrochemical workstation (Biologic, France).

The operando pressure test was operated on ECC-PRESS system (EL-cell GmbH, Germany) to quantify the consumed gas during the discharge process.

#### DEMS test

The DEMS test was conducted with Ar as the carrier gas on HPR40 (HIDEN ANALYTICAL, UK). To confirm that the head space of the cell could be filled by pure CO_2_ at the given time and gas speed, the battery was flowed with Ar for almost 12 h at a 0.4 ml/min velocity. And after this, the MS signals of the O_2_, N_2_ and CO_2_ were recorded. As we can see from Figure S1, during the first 2 hours, the signals are stable. Then the cell was opened to let air in and sealed quickly. It was clear that the signals for the three gases surged significantly and gradually decreased after sealing. When the signals returned to their previous values, we could deduce that the gas impurities were completely removed after the gas flowing in this period. From Figure S1, we observed that after 2 h, the head space was filled by pure Ar again. The result was consistent with calculation, which listed below.

Theoretically, we take the end when residue gas is less than 10 ppm. The dead volume of the ECC-Air is 4.2 ml while the flow rate is 0.4 ml/min.

So, after 1 min, the residue gas is 3.8 ml and the percentage is $\left(\frac{3.8}{4.2}\right)*100\%$;

After 2 min, the residue gas percentage is ${\left(\frac{3.8}{4.2}\right)}^{2}*100\%$;

After 3 min, the residue gas percentage is ${\left(\frac{3.8}{4.2}\right)}^{3}*100\%$;

…

We hypothesize that after N min, the residue gas is less than 10 ppm, we can get this in equation:

$${\left(\frac{3.8}{4.2}\right)}^{N}*100\%<10^{-5}$$

$$\text{Nlg}\frac{3.8}{4.2}<-5$$

N> 115

The result is N > 115. The 115 min matches the 2 h result from the experiment. In actual battery test, we set at least 3 h rest with flowing gas at a speed of 2 ml/min which possesses longer purge time and faster flow rate. So we can definitely ensure that the sealability of the Li-CO_2_ test can prevent any O_2_ permeation.

#### ^13^C isotope labeling test

To avoid carbon contamination, nanoporous gold (NPG) was used as the cathode in the Li-^13^CO_2_ battery. Due to the fragile NPG and rough surface of glass fiber separator, an additional PP separator was used at the cathode side to keep the integrity of NPG during battery assembly and test. After discharge, the PP membrane and NPG were directly immersed in D_2_O to dissolve the discharge products and intermediates. The solution was then ultrasonicated and centrifuged to get the upper clear solution for ^13^C NMR test based on AVANCE III 400MHz.

#### Physical characterization

The cathodes were washed with acetonitrile for 3 times followed by vacuum drying in the air lock chamber of the glove box. Raman spectroscopy experiments were conducted on a Horiba LabRAM HR Evolution confocal Raman microscope (T64000) with a 532-nm laser. The signals of the samples were collected 3 times with a collection time of 60 s using 50× lens. The XRD results were obtained from a MiniFlex 600 (Rigaku, Japan) X-ray diffractometer with a copper target. The SEM pictures were acquired on a field emission Hitachi S-4800 at a voltage of 10 kV and a current of 10 mA. FTIR spectra were performed on a Nicolet 6700 spectrometer (Thermo Scientific, US).

#### Calculation methods

In this work, all spin-polarized periodic density functional theory (DFT) calculations were carried out by the Vienna ab initio simulation package (VASP).^49^ The valence electron-ion interaction was described using the projector-augmented plane wave (PAW).^50^^,^^51^ A generalized gradient approximation (GGA) was expressed by the PBE functional.^52^ A 500 eV cutoff for the plane-wave basis set was adopted for optimizing primitive cell of graphite, and 400 eV cutoff with van der Waals (vDW) corrections was used in the remained calculations. We constructed a 5x5 supercell model for the Li-O_2_ and Li-CO_2_ reaction mechanisms. The vacuum space along the c direction was set to be 15 Å which was enough to avoid any interlayer interactions in the slab calculations. Integrations over the Brillouin zones were sampled with a 4x4x1 mesh of uniformly spaced *k* points for slabs and 11x11x11 mesh for primitive graphite. The convergence criterion of energy and force for structural optimization were set as 10^-5^ eV and 0.02 eV/Å.

The standard free energies (Δ $G_{f}^{{}^{\circ}}$) are calculated based on Equation 1

$$\Delta G_{f}^{{}^{\circ}}=\sum G_{\text{products}}^{{}^{\circ}}-\sum G_{\text{reactants}}^{{}^{\circ}}$$

(Equation 1) $$\Delta G_{f}^{{}^{\circ}}=\sum E_{\text{products}}-\sum E_{\text{reactants}}+\Delta E_{\text{ZPE}}-T\Delta S$$

where ∑*E*_products_ and ∑*E*_reactants_ are represented the total energy of products and reagents, respectively, and *E*_ZPE_ is the zero point energy, *T* is the reaction temperature (298.15K in this case), and *S* is the entropy.
