# Supplementary material for: The true performance of Li-CO2 batteries for resolving the disagreement on their feasibility
Source: iScience. 2025 Nov 14;28(12):114047. doi: 10.1016/j.isci.2025.114047 (PMC12686804; doi:10.1016/j.isci.2025.114047)
Supplement: Document S1. Figures S1–S16 and Table S1 [file mmc1.pdf]

iScience, Volume *28*

Supplemental information

The true performance of Li-CO_2 batteries for resolving the disagreement on their feasibility

Kai Chen, Jia-Yi Du, Hao-Ran Zhang, Ying-Qi Fan, Dong-Yue Yang, Jin Wang, Kai Li, and Gang Huang

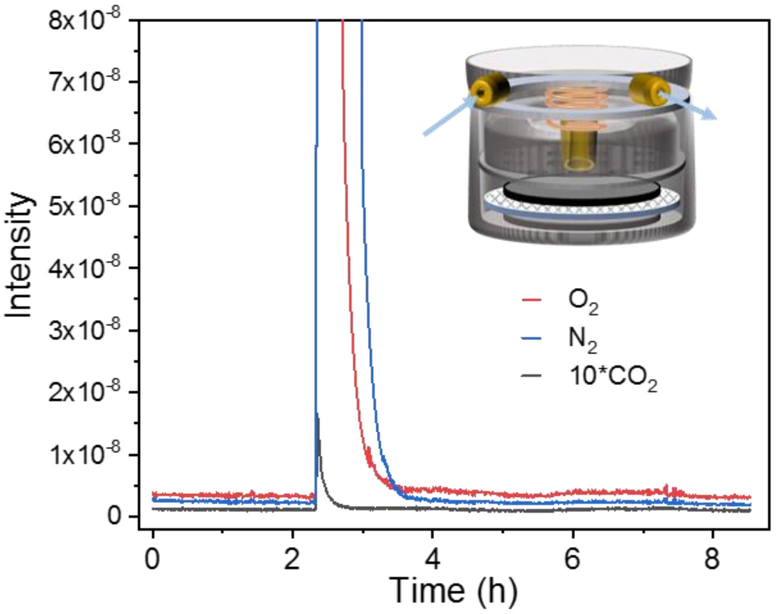

Figure S1. DEMS result of the experiment to confirm the time to remove the gas in the CELLs. The carrier gas is Ar (99.999%) with a 0.4 ml/min velocity.

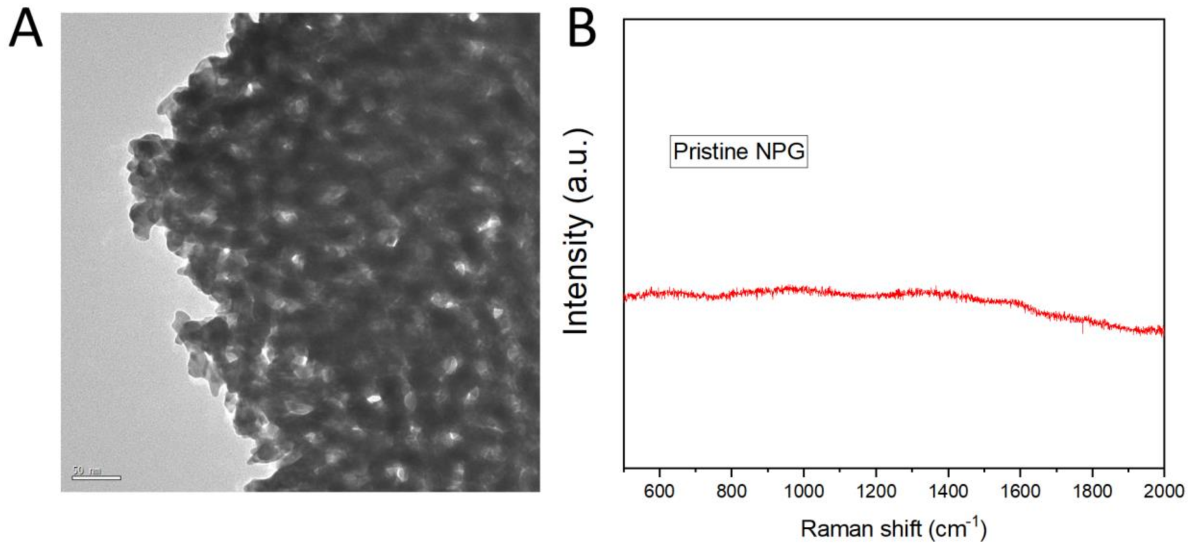

Fig. S2 Characterization of nanoporous Au (NPG). (A) TEM image of the NPG after dealloying AuAg by HNO_3. (B) Raman spectroscopy of pristine NPG.

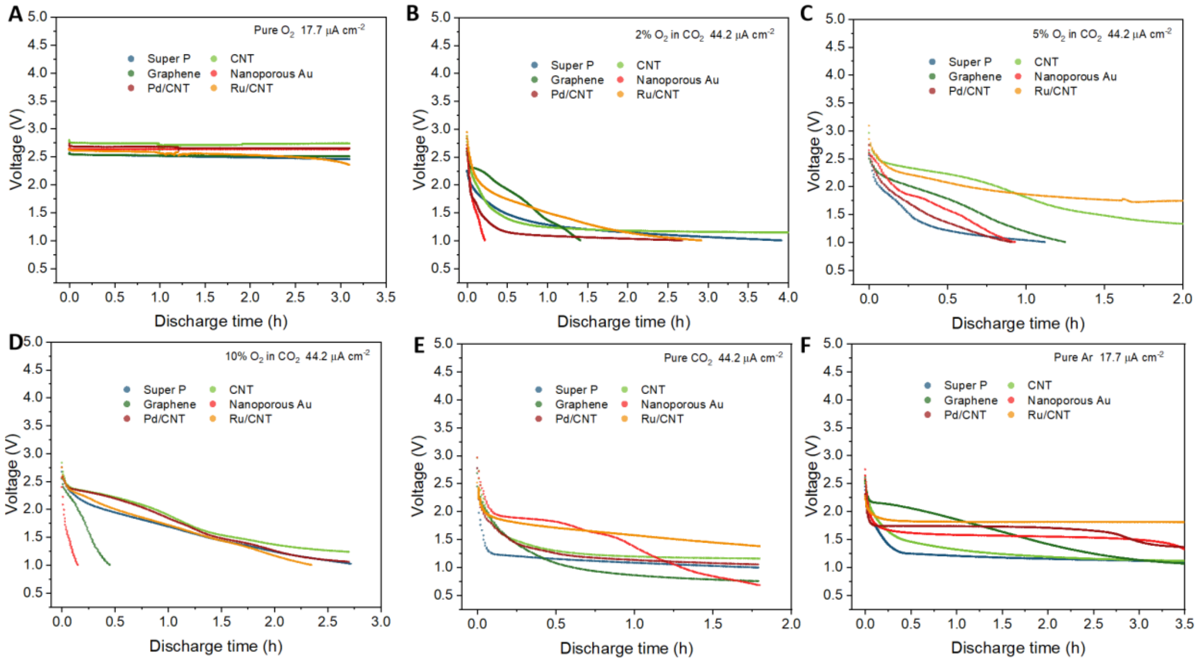

Fig. S3 Battery performance in different gas environments. (A) Discharge performance of true Li-O_2 batteries at 17.7 μA cm^{-2} and (B-E) Li-O_2/CO_2 batteries with different gas ratios and cathodes at 44.2 μA cm^{-2}. (F) Discharge curves of Li-based batteries with different catalysts in Ar at 17.7 μA cm^{-2}.

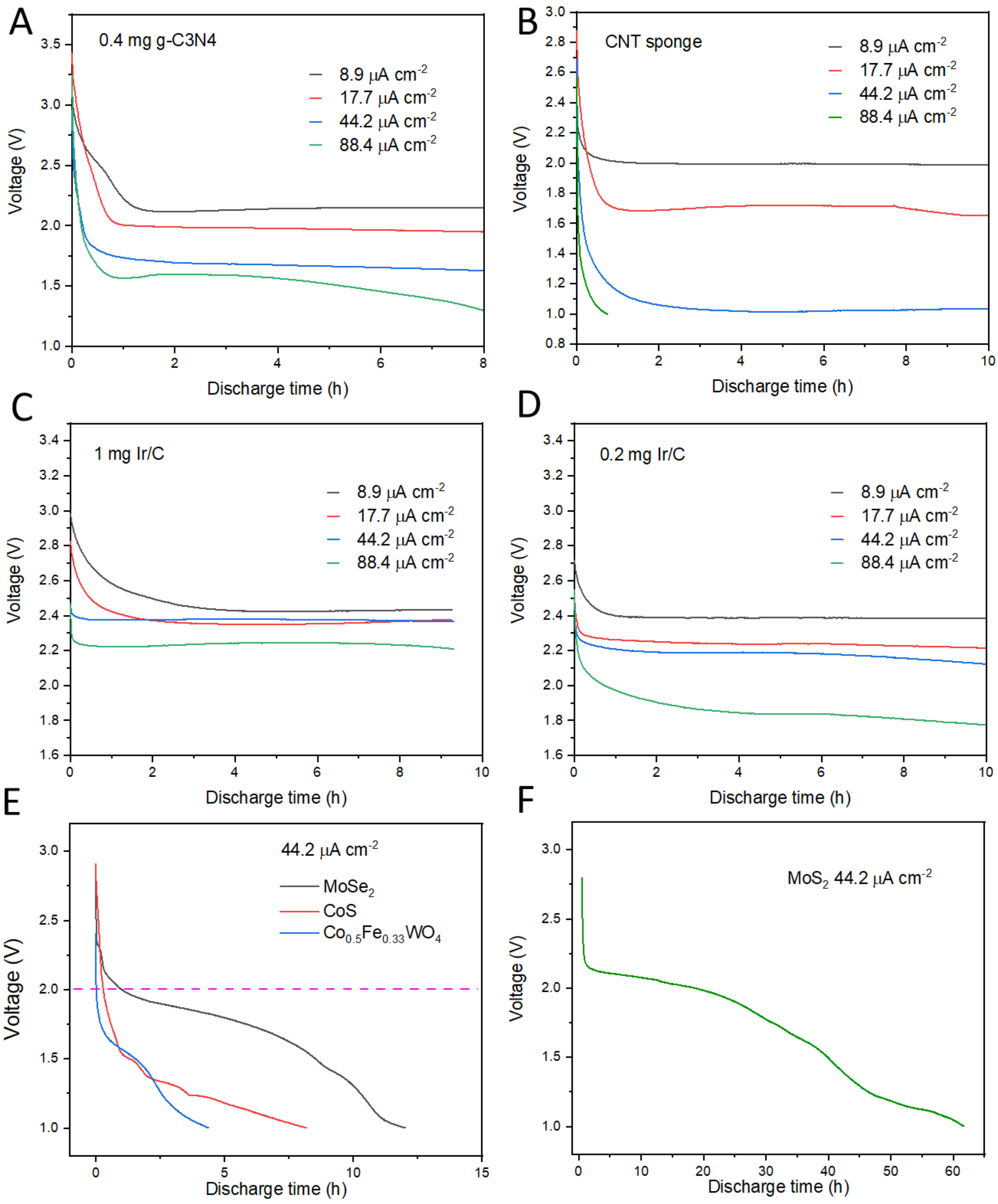

Fig. S4 Discharge performance of Li-CO_2 batteries with different cathode materials. The discharge curve of Li-CO_2 batteries with (A) g-C_3N_4, (B) CNT sponge, (C, D) Ir/C, (E) $MoSe_2$, CoS, $Co_{0.5}Fe_{0.33}WO_4$, and (F) MoS_2 cathodes.

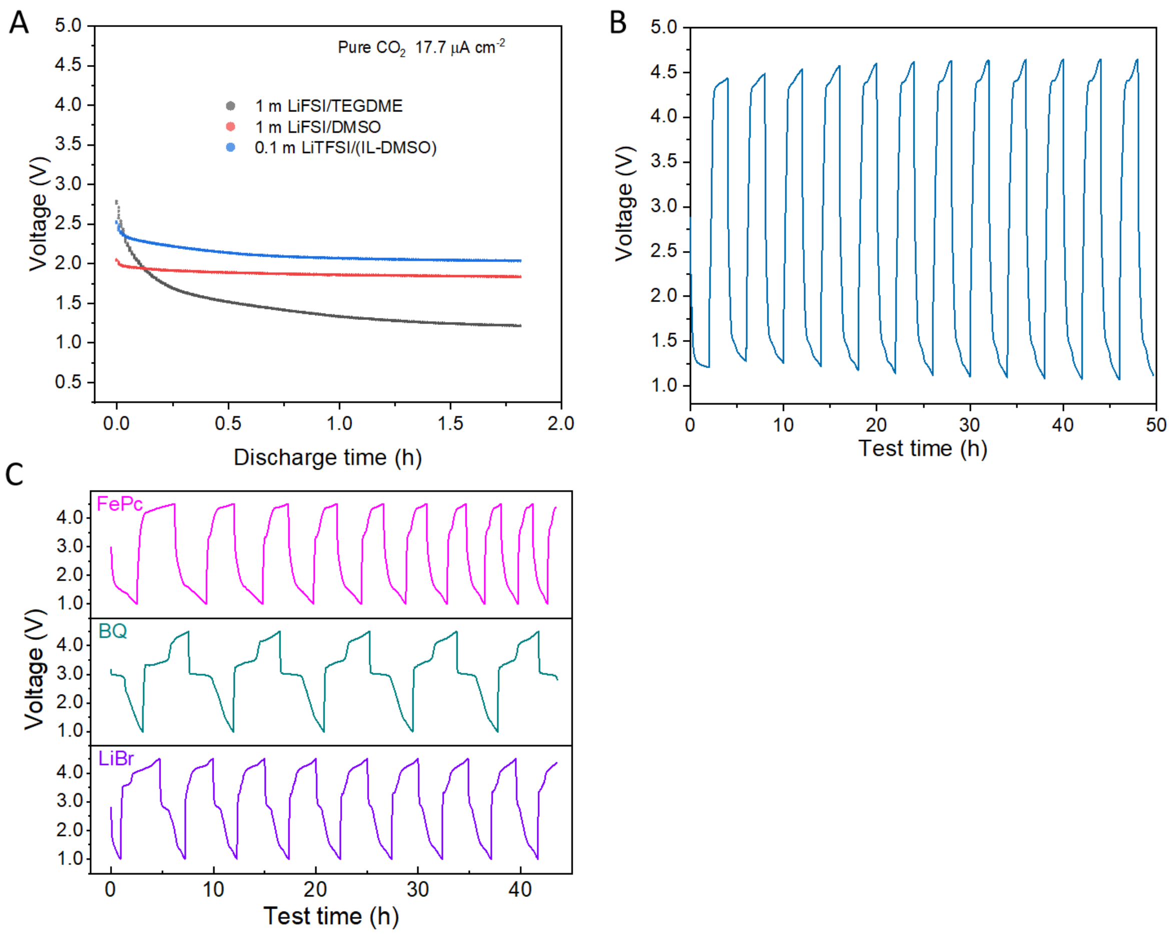

Fig. S5 Electrochemical performance of Li-CO_2 batteries with different compositions or at different test conditions. (A) Discharge performance of Li-CO_2 batteries at 17.7 μA cm^{-2} with different electrolytes. Cycling curves of CNT-based Li-CO_2 batteries (B) without RMs and (C) with 50 mM RMs (FePc= Iron phthalocyanine, BQ=Benquine, LiBr) in the electrolytes at 44.2 μA cm^{-2}.

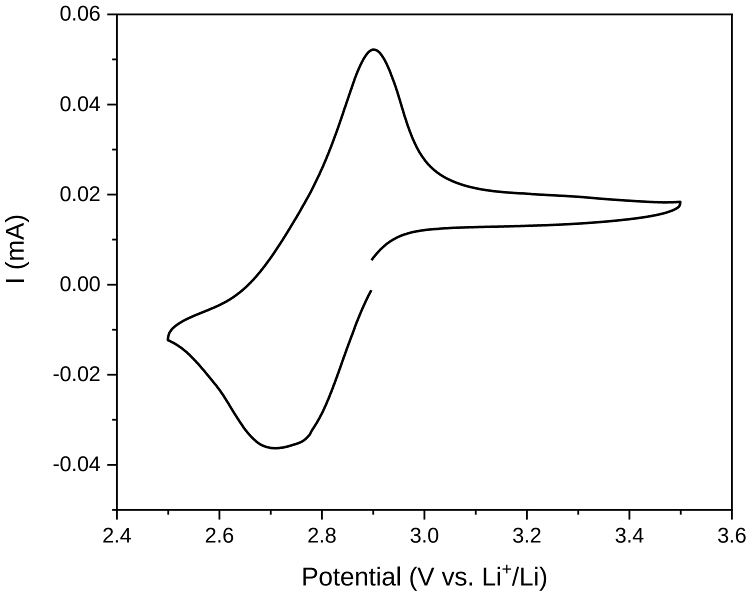

Fig. S6 CV curve of BQ in a Li-Ar battery between 2.5-3.5 V at a scan rate of 0.1 mV/s.

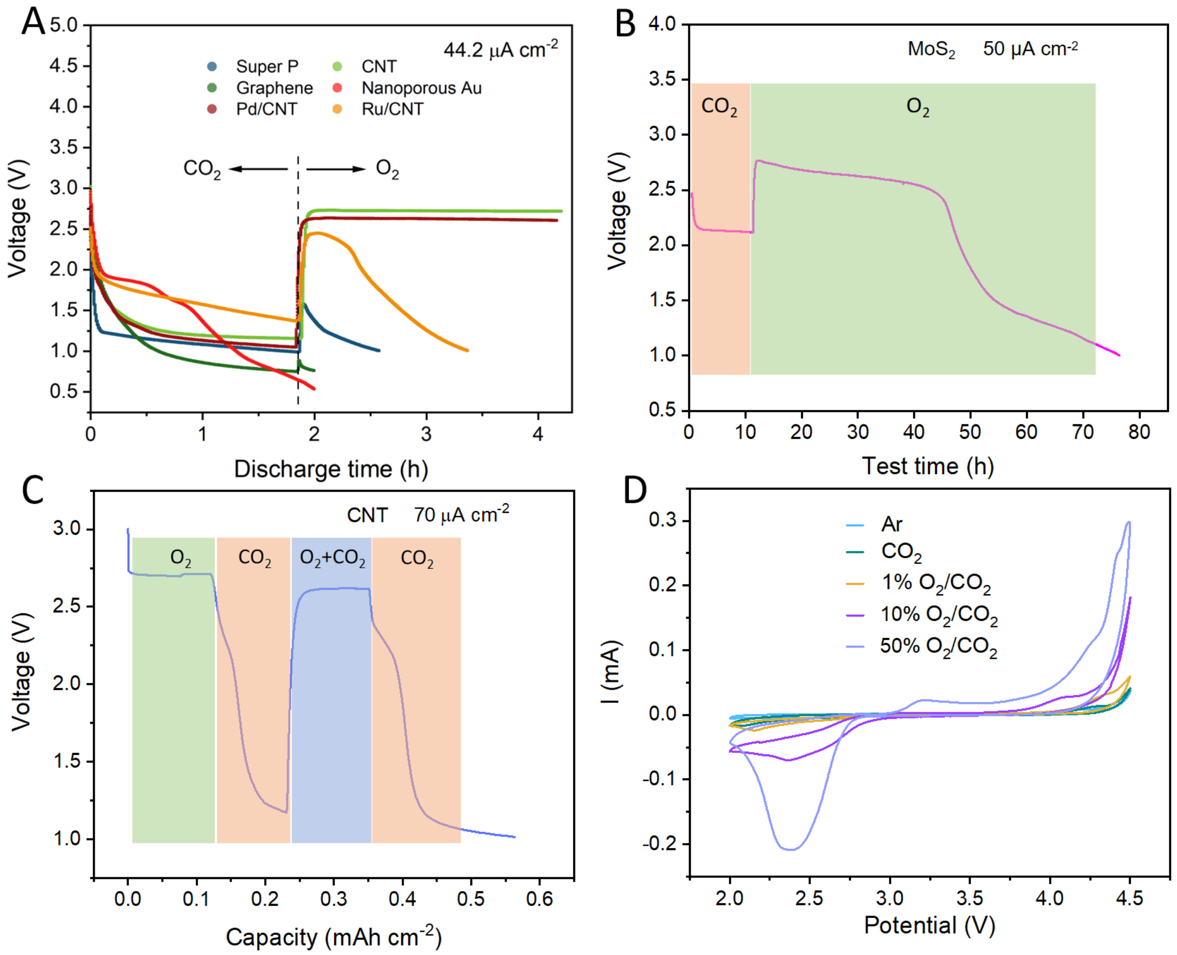

Fig. S7 The influence of O_2 in Li-CO_2 batteries. (A) O_2 purge in the original Li-CO_2 batteries. Gas switch of Li-O_2 and CO_2 batteries with (B) MoS_2 and (C) CNT cathodes. (D) CV curves of Li-CNT in different gas environments at a rate of 0.1 mV s^{-1} between 2.0-4.5 V.

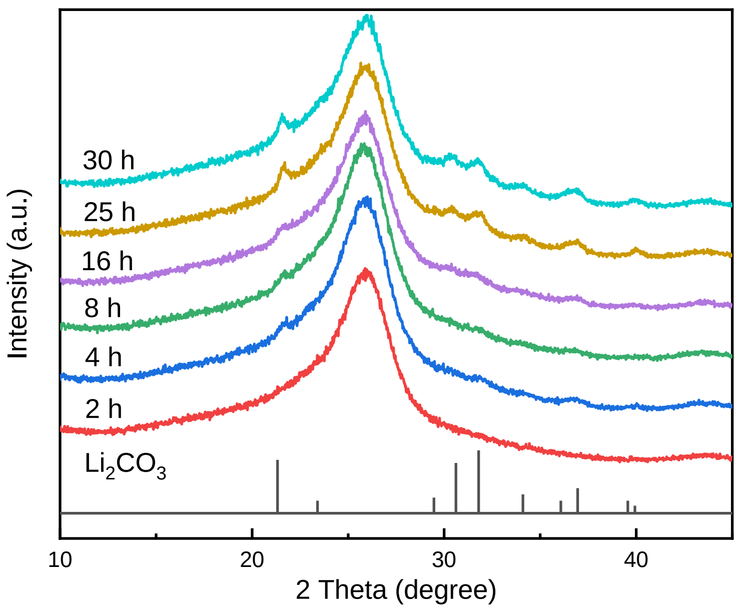

Fig. S8 XRD patterns of the CNT cathodes in Li-CO_2 batteries with different discharge time at 44.2 μA cm^{-2}.

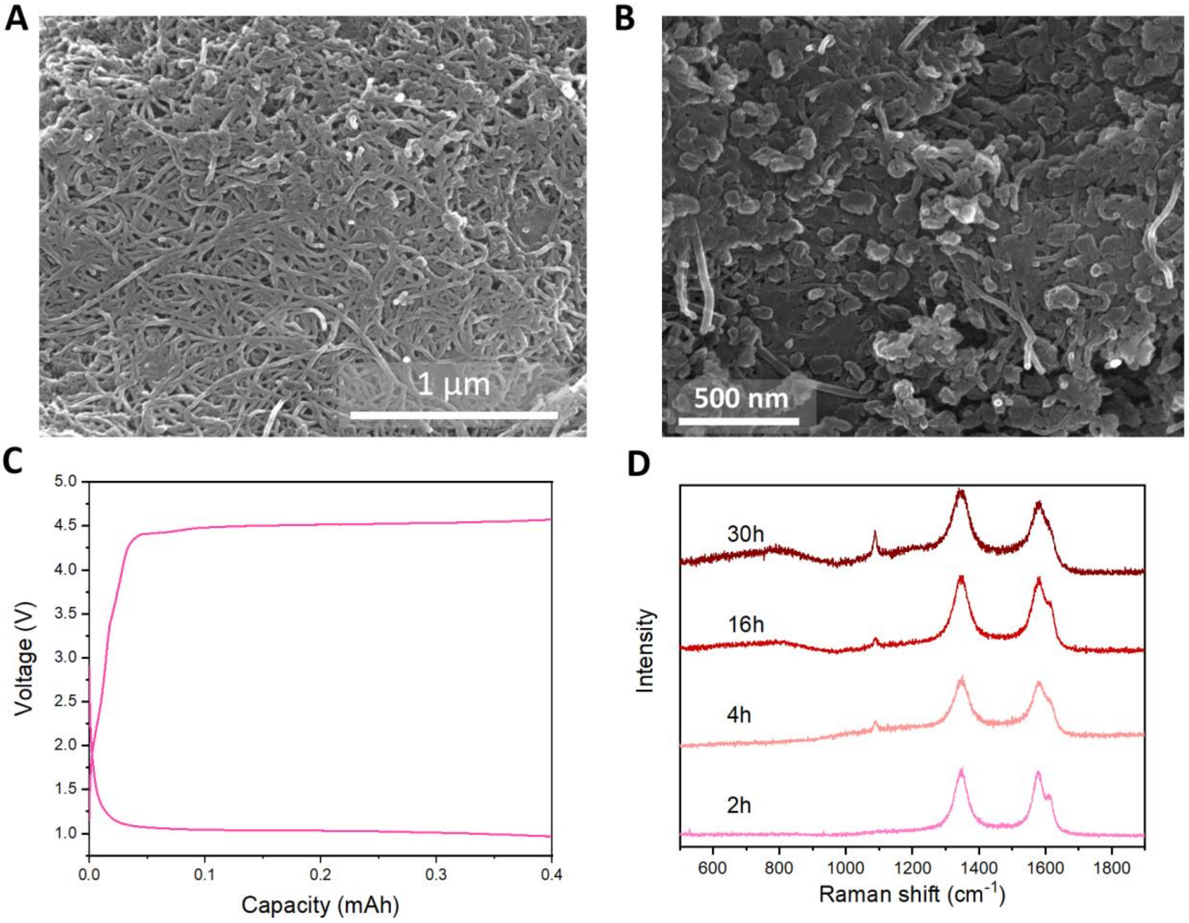

Fig. S9 Characterization of the discharge product of Li-CO_2 batteries. (A) Pristine and (B) discharged CNT cathodes. (C) The typical discharge and charge curves. (D) Raman spectra of the CNT cathodes after discharge for different time. Current density is 44.2 μA cm^{-2} (50 μA).

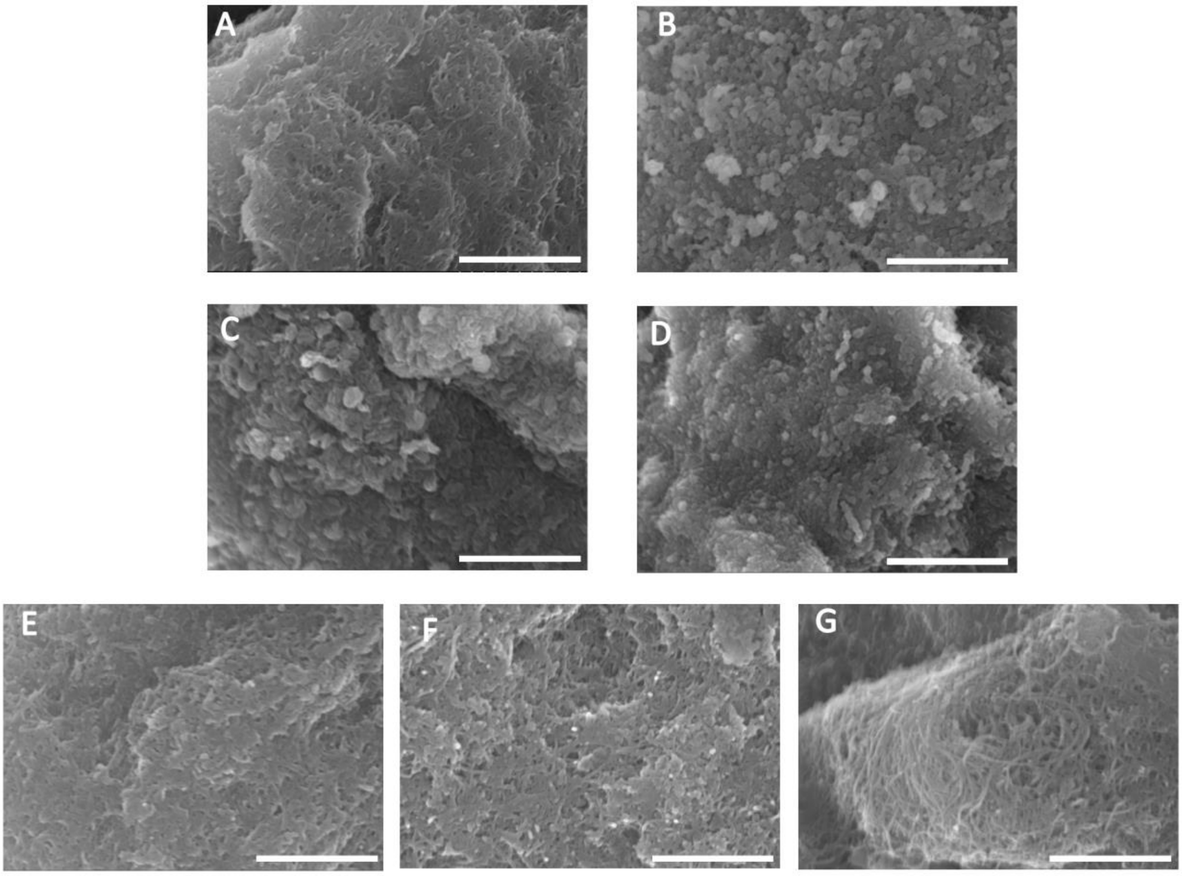

Fig. S10 Discharge product morphologies. SEM images of the cathodes in Li-CO_2 batteries after discharge for (A) 2 h, (B) 4 h, (C) 8 h and (D) 16 h, and charge for (E) 4 h, (F) 8 h and (G) 16 h at 50 μA. Scale bar is 2 μm.

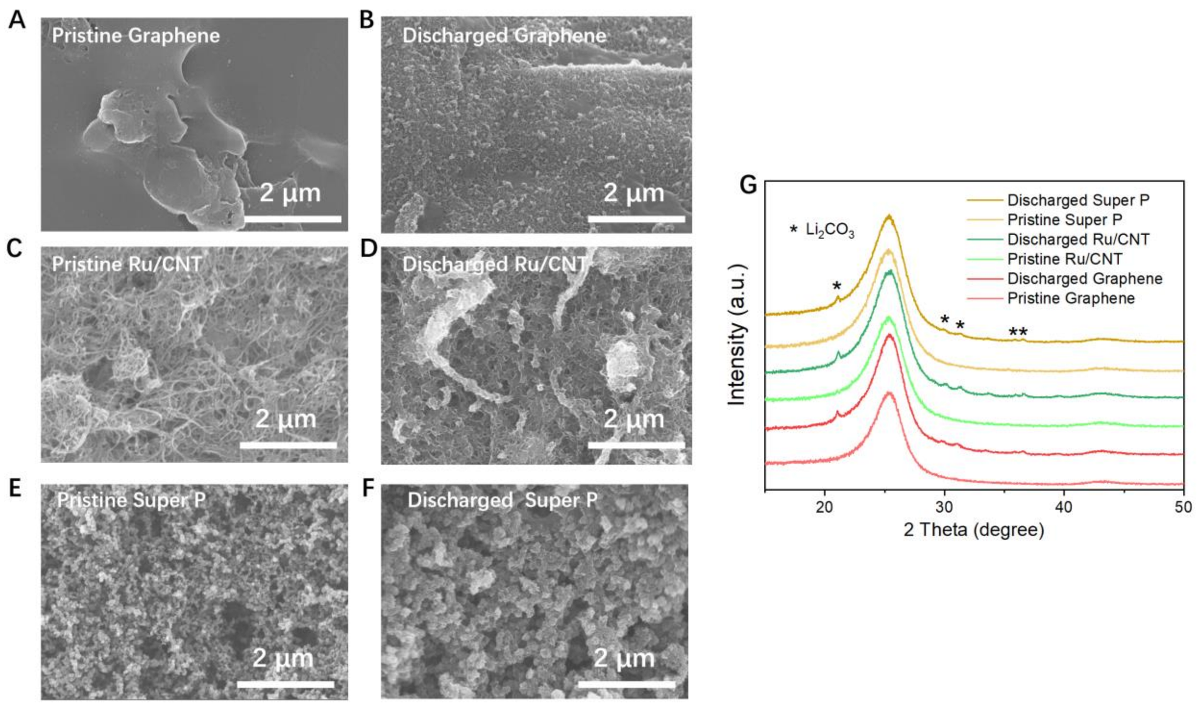

Fig. S11. Characterization of discharged cathodes. The morphologies of (A,B) graphene, (C,D) Ru/CNT, and (E,F) Super P before and after discharge in Li-CO_2 batteries. (G) XRD patterns of the discharged catalysts.

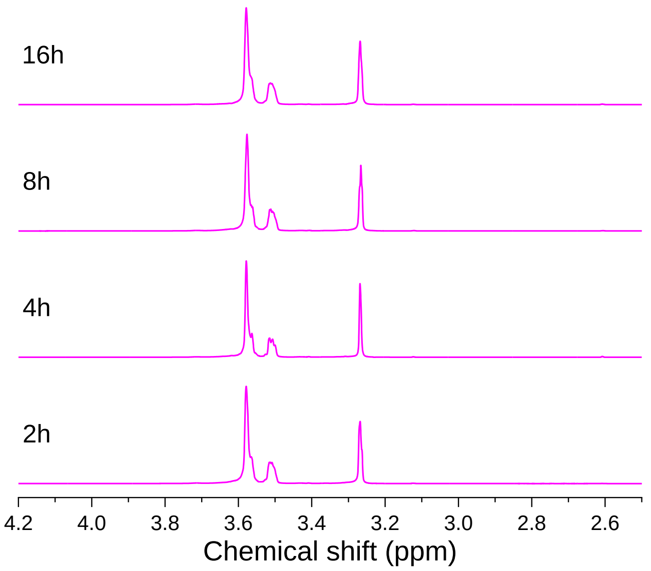

Fig. S12 ^{1}H NMR spectra of the electrolytes after discharge in Li-CO_2 batteries at 50 μA.

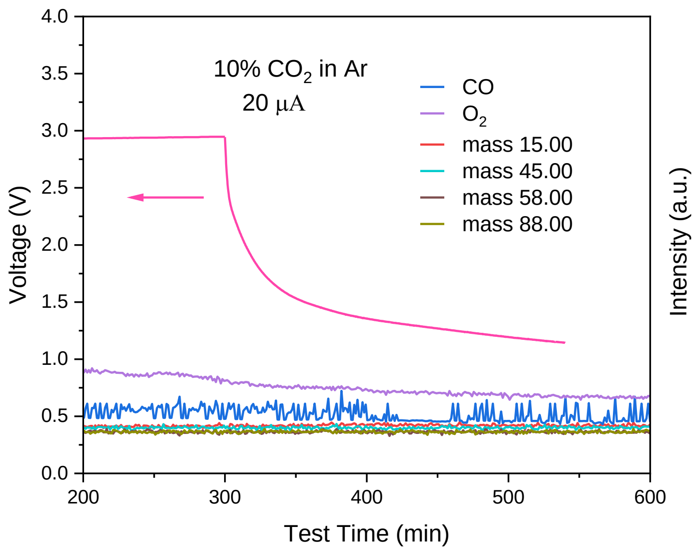

Fig. S13 DEMS test during discharge of the Li-CO_2 battery at 20 μA.

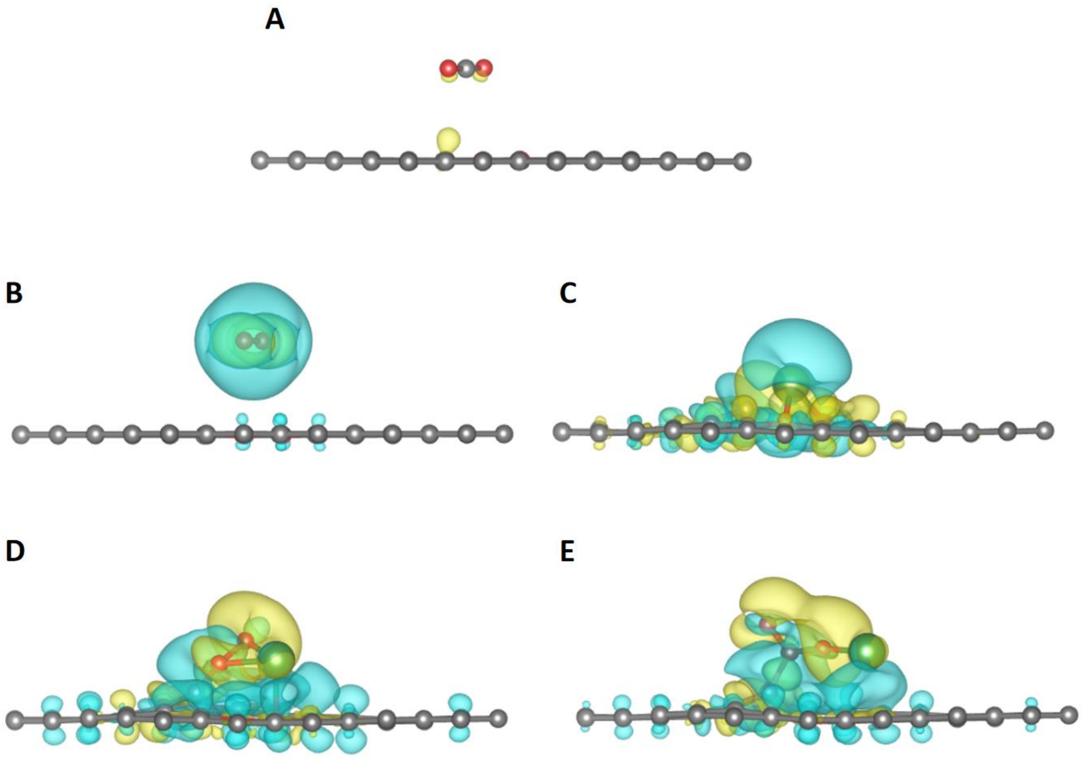

Fig. S14 Charge density difference plots. Charge density difference of (a) CO_2, (b) O_2 and (c) Li^+ absorbed on the CNT (002) surface, and (d) O_2 and (e) CO_2 absorbed on *Li. The isosurface value of charge density difference distribution is set to be 0.001 e/Bohr^3 for CNT, and the yellow and light blue regions represent the charge accumulation and charge loss, respectively.

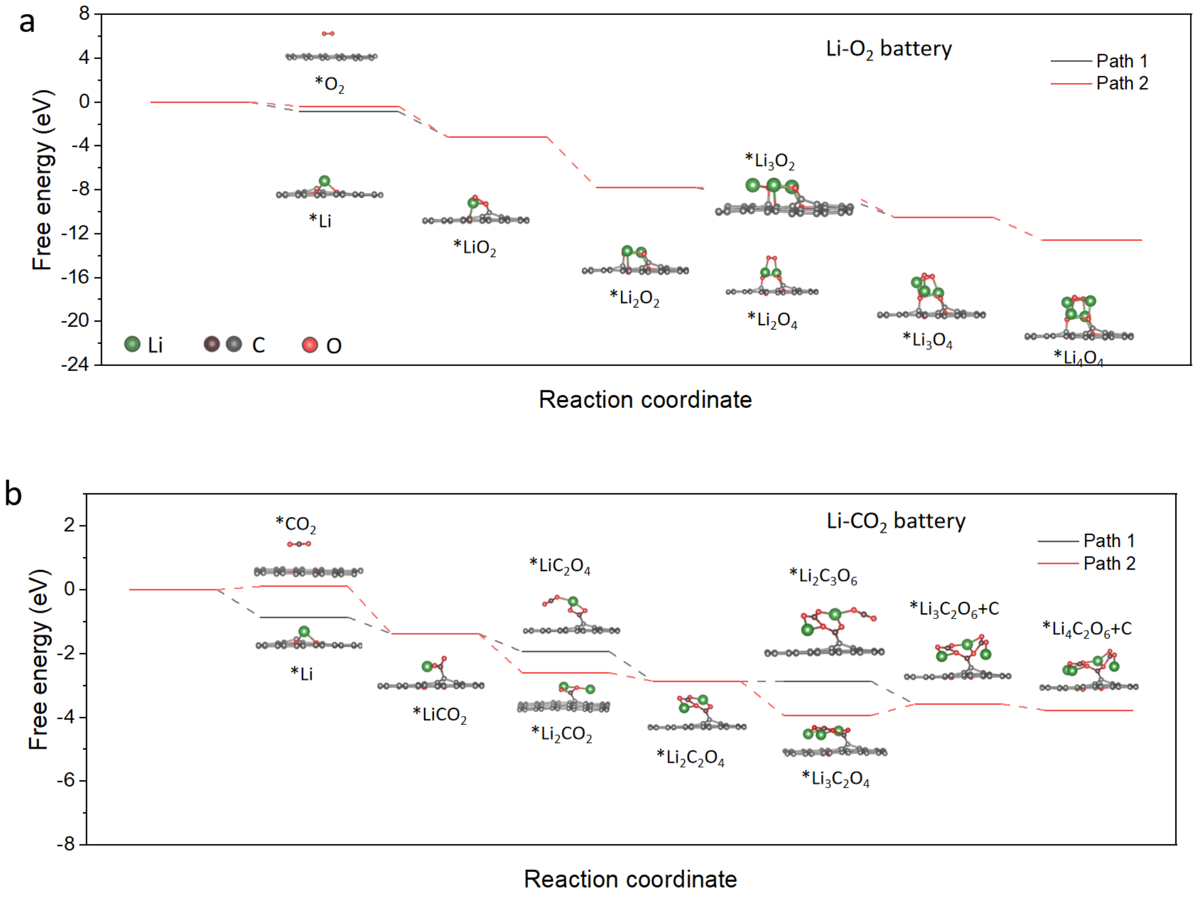

Fig. S15 Discharge paths of Li-O_2 or -CO_2 batteries. Proposed reaction intermediates of the Li-O_2 (a) and Li-CO_2 batteries(b) during the discharge process.

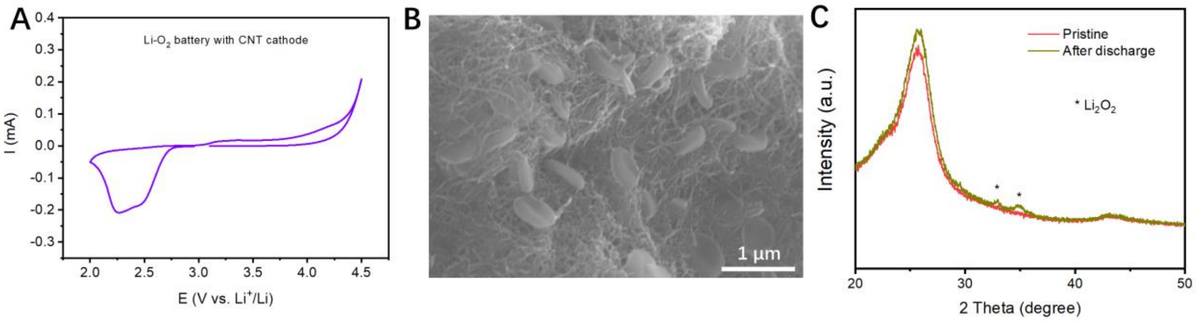

Fig. S16. Characterization of discharge product of Li-O_2 batteries. (a) Cyclic voltammetry of Li-O_2 battery with CNT cathode and corresponding (b)SEM; (c) XRD pattern after discharge.

A Table S1. A summary of the reported Li-CO_2 batteries.

Reference	Cathode	Electrolyte	Current density (mA g^{-1})	Discharge plateau (V)	Capacity (mAh g^{-1})	Carbon formation test	GITT test	Mass spectra to ensure gas tight
Liu et al. (2014)[1]	KB	$LiCF_3SO_3$/TEGDME=1:4	30	2.7	1050	Yes	No	No
Zhang et al. (2015)[2]	CNT	1 m LiTFSI/TEGDME	50	2.7	8379	No	No	No
Zhang et al. (2015)[3]	Graphene	1 m LiTFSI/TEGDME	50	2.77	14722	Yes	No	No
Li et al. (2017)[4]	CNT	Gel polymer electrolyte	50	2.7	8536	No	No	No
Qiao et al. (2017)[5]	Sputtered gold cathode	0.5 m $LiClO_4$/DMSO	5 μA	2.5 V	>10 μAh	Yes	No	No
Qie et al. (2017)[6]	B,N-holy graphene	1 m LiTFSI/TEGDME	300	2.8 V	16033	No	No	No
Yang et al. (2017)[7]	Ru@Super P	$LiCF_3SO_3$/TEGDME=1:4	100	2.75	8229	Yes	No	No
Wang et al. (2017)[8]	KB	1 m LiTFSI/TEGDME with 0.05 m LiBr	200	2.5	>500	No	No	No
Zhu et al. (2017)[9]	MoFeNi&MoC @N-doped carbon	1 m LiTFSI/TEGDME	100	2.83	8827	No	No	No
Hu et al. (2017)[10]	CNT	Composite polymer electrolyte	100	2.3	>500	No	No	No
Yin et al. (2018)[11]	Super P	0.1 m $LiClO_4$/MeCN with 0.005 m DBBQ	N/A	2.4	160	No	No	No
Li et al. (2018)[12]	Mn_2(dobdc)	1 m LiTFSI/TEGDME	50	2.7	18022	No	No	No
Pipes et al. (2018)[13]	TiO_2 NP@CNT/CNF	1 m LiTFSI/DMSO	50 μA cm^{-2}	2.8	1950 μAh cm^{-2}	No	No	No
Xu et al. (2018)[14]	Ru/CNT on wood	1 m LiTFSI/TEGDME	100	2.7	N/A	No	No	No
Xu et al. (2018)[15]	RuO_2/LDO	1 m LiTFSI/TEGDME	166	2.5	1000	No	No	No
Zhang et al. (2018)[16]	NiO–CNT	1 m LiTFSI/TEGDME	100	2.7	9000	No	No	No
Zhang et al. (2018)[17]	Ni-NG	1 m LiTFSI/TEGDME	100	2.7	17625	No	No	No
Zhang et al. (2018)[18]	Cu-NG	1 m LiTFSI/TEGDME	200	2.8	14864	No	No	No
Jin et al. (2018)[19]	CQD/hG-0.3	(1 m LiTFSI + 0.3 m $LiNO_3$)/DMSO	500	2.8	12300	No	No	No
Xing et al. (2018)[20]	Ir NSs-CNFs	1 m LiTFSI/TEGDME	0.05 mA cm^{-2}	2.8	2.3 mAh cm^{-2}	Yes	No	No
Alireza et al. (2019)[21]	MoS_2 nanoflakes	0.1 m (EMIM-BF_4)/DMSO (25%/75%)	500	2.9	57500	Yes	No	No
Bie et al. (2019)[22]	Ru@CNT	$LiCF_3SO_3$/TEGDME=1:4	50	2.5	3874	No	No	No
Guo et al. (2019)[23]	RuP_2-NPCFs	1 m LiTFSI/TEGDME	100	2.7	11951	Yes	No	No
Aliza et al. (2019)[24]	Vulcan carbon	1 m $LiCF_3SO_3$/TEGDME	30	2.68	1992	No	No	No
Li et al. (2019)[25]	MnO@NC-rGO	1 m LiTFSI/TEGDME	50	3.0	25021	No	No	No
Li et al. (2019)[26]	COF-Ru@CNT	1 m LiTFSI/TEGDME	200	2.75	27348	No	No	No
Li et al. (2019)[27]	B-NCNT	1 m LiTFSI/TEGDME	50	2.5	23328	No	Eeq=3.1 V	No
Li et al. (2019)[28]	N-CNTs@Ti	1 m LiTFSI/TEGDME	50	2.65	9292.3	No	No	No

Reference	Cathode	Electrolyte	Current density (mA g^{-1})	Discharge plateau (V)	Capacity (mAh g^{-1})	Carbon formation test	GITT test	Mass spectra to ensure gas tight
Zhang et al. (2019)[29]	Ru-Cu-G	1 m LiTFSI/TEGDME	200	2.6	14000	No	No	No
Zhou et al. (2019)[30]	Mo_2C@CNT	Gel polymer electrolyte	50 μA cm^{-2}	2.7	3415 μAh cm^{-2}	No	No	No
Song et al. (2019)[31]	Co-N-CNT	(1 m LiTFSI + 0.3 m $LiNO_3$)/DMSO	200	2.8	6042	No	No	No
Qiao et al. (2019)[32]	Ru/ACNF	1 m LiTFSI/TEGDME	100	2.8	N/A	No	No	No
Xiao et al. (2020)[33]	CNT	1 m LiTFSI/TEGDME	150	2.6	> 1000	No	No	No
Huang et al. (2019)[34]	Graphene@COF	1 m LiTFSI/TEGDME	75	2.3	27833	No	No	No
Hu et al. (2020)[35]	Fe-ISA/N,S-HG	(1 m LiTFSI + 0.3 m $LiNO_3$)/DMSO	100	2.75	23174	No	No	No
Li et al. (2020)[36]	Ir/C	1 m LiTFSI/DOL	100	2.5	14720	No	No	No
Li et al. (2020)[37]	MnO@NMCNF	1 m LiTFSI/TEGDME	0.04 mA cm^{-2}	2.85	19.07 mAh cm^{-2}	No	No	No
Wu et al. (2020)[38]	IrO_2-N/CNT	1 m LiTFSI/TEGDME	100	2.6	4634	No	No	No
Xiao et al. (2020)[39]	3D NCNT/G	(1 m LiTFSI + 0.3 m $LiNO_3$)/DMSO	N/A	2.7	17534.1	No	No	No
Guan et al. (2020)[40]	In_2S_3@CNT/SS	1 m LiTFSI/TEGDME	10 μA cm^{-2}	2.73	N/A	No	No	No
Li et al. (2020)[41]	SiC/RGO	1 m LiTFSI/TEGDME	20	2.5	1602	No	No	No

References

1. Liu, Y., Wang, R., Lyu, Y., Li, H., and Chen, L. (2014). Rechargeable Li/CO_2-O_2 (2 : 1) battery and Li/CO_2 battery. Energy Environ. Sci. *7*, 677-681.
2. Zhang, X., Zhang, Q., Zhang, Z., Chen, Y., Xie, Z., Wei, J., and Zhou, Z. (2015). Rechargeable Li-CO_2 batteries with carbon nanotubes as air cathodes. Chem. Commun. *51*, 14636-14639.
3. Zhang, Z., Zhang, Q., Chen, Y., Bao, J., Zhou, X., Xie, Z., Wei, J., and Zhou, Z. (2015). The First Introduction of Graphene to Rechargeable Li-CO_2 Batteries. Angew. Chem. Int. Ed. *54*, 6550-6553.
4. Li, C., Guo, Z., Yang, B., Liu, Y., Wang, Y., and Xia, Y. (2017). A rechargeable Li-CO_2 battery with a gel polymer electrolyte. Angew. Chem. Int. Ed. *56*, 9126-9130.
5. Qiao, Y., Yi, J., Wu, S., Liu, Y., Yang, S., He, P., and Zhou, H. (2017). Li-CO_2 Electrochemistry: A New Strategy for CO_2 Fixation and Energy Storage. Joule *1*, 359-370.
6. Qie, L., Lin, Y., Connell, J.W., Xu, J., and Dai, L. (2017). Highly rechargeable lithium-CO_2 batteries with a boron- and nitrogen-codoped holey-graphene cathode. Angew. Chem. Int. Ed. *129*, 7074-7078.
7. Yang, S., Qiao, Y., He, P., Liu, Y., Cheng, Z., Zhu, J.-j., and Zhou, H. (2017). A reversible lithium-CO_2 battery with Ru nanoparticles as a cathode catalyst. Energy Environ. Sci. *10*, 972-978.
8. Wang, X.-G., Wang, C., Xie, Z., Zhang, X., Chen, Y., Wu, D., and Zhou, Z. (2017). Improving electrochemical performances of rechargeable Li-CO_2 batteries with an electrolyte eedox mediator. ChemElectroChem *4*, 2145-2149.

9. Zhu, Q.-C., Xu, S.-M., Cai, Z.-P., Harris, M.M., Wang, K.-X., and Chen, J.-S. (2017). Towards real Li-air batteries: A binder-free cathode with high electrochemical performance in CO_2 and O_2. Energy Storage Mater. *7*, 209-215.
10. Hu, X., Li, Z., and Chen, J. (2017). Flexible Li-CO_2 batteries with liquid-free electrolyte. Angew. Chem. Int. Ed. *56*, 5785-5789.
11. Yin, W., Grimaud, A., Azcarate, I., Yang, C., and Tarascon, J.-M. (2018). Electrochemical reduction of CO_2 mediated by quinone derivatives: Implication for Li-CO_2 battery. J. Phys. Chem. C *122*, 6546-6554.
12. Li, S., Dong, Y., Zhou, J., Liu, Y., Wang, J., Gao, X., Han, Y., Qi, P., and Wang, B. (2018). Carbon dioxide in the cage: manganese metal–organic frameworks for high performance CO_2 electrodes in Li-CO_2 batteries. Energy Environ. Sci. *11*, 1318-1325.
13. Pipes, R., Bhargav, A., and Manthiram, A. (2018). Nanostructured anatase titania as a cathode catalyst for Li-CO_2 batteries. ACS Appl. Mater. Interfaces *10*, 37119-37124.
14. Xu, S., Chen, C., Kuang, Y., Song, J., Gan, W., Liu, B., Hitz, E.M., Connell, J.W., Lin, Y., and Hu, L. (2018). Flexible lithium-CO_2 battery with ultrahigh capacity and stable cycling. Energy Environ. Sci. *11*, 3231-3237.
15. Xu, S.M., Ren, Z.C., Liu, X., Liang, X., Wang, K.X., and Chen, J.S. (2018). Carbonate decomposition: Low-overpotential Li-CO_2 battery based on interlayer-confined monodisperse catalyst. Energy Storage Mater. *15*, 291-298.
16. Zhang, X., Wang, C., Li, H., Wang, X.-G., Chen, Y.-N., Xie, Z., and Zhou, Z. (2018). High performance Li-CO_2 batteries with NiO-CNT cathodes. J. Mater. Chem. A *6*, 2792-2796.
17. Zhang, Z., Wang, X.-G., Zhang, X., Xie, Z., Chen, Y.-N., Ma, L., Peng, Z., and Zhou, Z. (2018). Verifying the rechargeability of Li-CO_2 batteries on working cathodes of Ni nanoparticles highly dispersed on N-doped graphene. Adv. Sci. *5*, 1700567.
18. Zhang, Z., Zhang, Z., Liu, P., Xie, Y., Cao, K., and Zhou, Z. (2018). Identification of cathode stability in Li-CO_2 batteries with Cu nanoparticles highly dispersed on N-doped graphene. J. Mater. Chem. A *6*, 3218-3223.
19. Jin, Y., Hu, C., Dai, Q., Xiao, Y., Lin, Y., Connell, J.W., Chen, F., and Dai, L. (2018). High-performance Li-CO_2 batteries based on metal-free carbon quantum dot/holey graphene composite catalysts. Adv. Funct. Mater. *28*, 1804630.
20. Xing, Y., Yang, Y., Li, D., Luo, M., Chen, N., Ye, Y., Qian, J., Li, L., Yang, D., Wu, F., et al. (2018). Crumpled Ir nanosheets fully covered on porous carbon nanofibers for long-life rechargeable lithium-CO_2 batteries. Adv. Mater. *30*, 1803124.
21. Ahmadiparidari, A., Warburton, R.E., Majidi, L., Asadi, M., Chamaani, A., Jokisaari, J.R., Rastegar, S., Hemmat, Z., Sayahpour, B., Assary, R.S., et al. (2019). A long-cycle-life lithium-CO_2 battery with carbon neutrality. Adv. Mater. *31*, 1902518.
22. Bie, S., Du, M., He, W., Zhang, H., Yu, Z., Liu, J., Liu, M., Yan, W., Zhou, L., and Zou, Z. (2019). Carbon nanotube@RuO_2 as a high performance catalyst for Li-CO_2 batteries. ACS Appl. Mater. Interfaces *11*, 5146-5151.
23. Guo, Z., Li, J., Qi, H., Sun, X., Li, H., Tamirat, A.G., Liu, J., Wang, Y., and Wang, L. (2019). A highly reversible long-life Li-CO_2 battery with a RuP_2-based catalytic cathode. Small *15*, 1803246.
24. Khurram, A., Yin, Y., Yan, L., Zhao, L., and Gallant, B.M. (2019). Governing role of solvent on discharge activity in lithium-CO_2 batteries. J. Phys. Chem. Lett. *10*, 6679-6687.

25. Li, S., Liu, Y., Zhou, J., Hong, S., Dong, Y., Wang, J., Gao, X., Qi, P., Han, Y., and Wang, B. (2019). Mono-dispersed MnO nanoparticles in graphene-interconnected N-doped 3D carbon framework as highly efficient gas cathode in Li-CO_2 batteries. Energy Environ. Sci. *12*, 1046-1054.
26. Li, X., Wang, H., Chen, Z., Xu, H.S., Yu, W., Liu, C., Wang, X., Zhang, K., Xie, K., and Loh, K.P. (2019). Covalent-organic-framework-based Li-CO_2 batteries. Adv. Mater. *31*, 1905879.
27. Li, X., Zhou, J., Zhang, J., Li, M., Bi, X., Liu, T., He, T., Cheng, J., Zhang, F., Li, Y., et al. (2019). Bamboo-like nitrogen-doped carbon nanotube forests as durable metal-free catalysts for self-powered flexible Li-CO_2 batteries. Adv. Mater. *31*, 1903852.
28. Li, Y.C., Zhou, J.W., Zhang, T.B., Wang, T.S., Li, X.L., Jia, Y.F., Cheng, J.L., Guan, Q., Liu, E.Z., Peng, H.S., et al. (2019). Highly surface-wrinkled and N-doped CNTs anchored on metal wire: A novel fiber-shaped cathode toward high-performance flexible Li-CO_2 batteries. Adv. Funct. Mater. *29*, 808117.
29. Zhang, Z., Yang, C., Wu, S., Wang, A., Zhao, L., Zhai, D., Ren, B., Cao, K., and Zhou, Z. (2019). Exploiting synergistic effect by integrating ruthenium-copper nanoparticles highly co-dispersed on graphene as efficient air cathodes for Li-CO_2 batteries. Adv. Energy Mater. *9*, 1802805.
30. Zhou, J., Li, X., Yang, C., Li, Y., Guo, K., Cheng, J., Yuan, D., Song, C., Lu, J., and Wang, B. (2019). A quasi-solid-state flexible fiber-shaped Li-CO_2 battery with low overpotential and high energy efficiency. Adv. Mater. *31*, 1804439.
31. Song, L., Wang, T., Wu, C., Fan, X., and He, J. (2019). A long-life Li-CO_2 battery employing a cathode catalyst of cobalt-embedded nitrogen-doped carbon nanotubes derived from a Prussian blue analogue. Chem. Commun. *55*, 12781-12784.
32. Qiao, Y., Xu, S., Liu, Y., Dai, J., Xie, H., Yao, Y., Mu, X., Chen, C., Kline, D.J., Hitz, E.M., et al. (2019). Transient, in situ synthesis of ultrafine ruthenium nanoparticles for a high-rate Li-CO_2 battery. Energy Environ. Sci. *12*, 1100-1107.
33. Xiao, X., Tan, P., Zhu, X., Dai, Y., Cheng, C., and Ni, M. (2020). Investigation on the discharge and charge behaviors of Li-CO_2 batteries with carbon nanotube electrodes. ACS Sustainable Chem. Eng. *8*, 9742-9750.
34. Huang, S., Chen, D., Meng, C., Wang, S., Ren, S., Han, D., Xiao, M., Sun, L., and Meng, Y. (2019). CO_2 nanoenrichment and nanoconfinement in cage of imine covalent organic frameworks for high-performance CO_2 cathodes in Li-CO_2 batteries. Small *15*, 1904830.
35. Hu, C., Gong, L., Xiao, Y., Yuan, Y., Bedford, N.M., Xia, Z., Ma, L., Wu, T., Lin, Y., Connell, J.W., et al. (2020). High-performance, long-life, rechargeable Li-CO_2 batteries based on a 3D holey graphene cathode implanted with dingle iron atoms. Adv. Mater. *32*, 1907436.
36. Li, J., Wang, L., Zhao, Y., Li, S., Fu, X., Wang, B., and Peng, H. (2020). Li-CO_2 batteries efficiently working at ultra-low temperatures. Adv. Funct. Mater. *30*, 2001619.
37. Li, S., Liu, Y., Gao, X., Wang, J., Zhou, J., Wang, L., and Wang, B. (2020). Improving areal capacity of flexible Li-CO_2 batteries by constructing freestanding cathode with monodispersed MnO nanoparticles in N-doped mesoporous carbon nanofibers. J. Mater. Chem. A *8*, 10354-10362.
38. Wu, G., Li, X., Zhang, Z., Dong, P., Xu, M., Peng, H., Zeng, X., Zhang, Y., and Liao, S. (2020). Design of an ultralong-life Li-CO_2 batteries with IrO_2 nanoparticles highly dispersed onto nitrogen-doped Carbon Nanotubes. J. Mater. Chem. A *8*, 3763-3770.
39. Xiao, Y., Du, F., Hu, C.G., Ding, Y., Wang, Z.L., Roy, A., and Dai, L.M. (2020). High-performance Li-CO_2 batteries from free-standing, binder-free, bifunctional three-dimensional carbon catalysts. ACS Energy Lett. *5*, 916-921.

40. Guan, D.H., Wang, X.X., Li, M.L., Li, F., Zheng, L.J., Huang, X.L., and Xu, J.J. (2020). Light/electricity energy conversion and storage for a hierarchical porous In_2S_3@CNT/SS cathode towards a flexible Li-CO_2 battery. Angew. Chem. Int. Ed. *59*, 19518-19524.
41. Li, Z., Li, M., Wang, X., Guan, D., Liu, W., and Xu, J.-J. (2020). In-situ fabricated photo-electro-catalytic hybrid cathode for light-assisted lithium-CO_2 batteries. J. Mater. Chem. A *8*, 14799-14806.
